# A plant host, *Nicotiana benthamiana*, enables the production and study of fungal lignin-degrading enzymes

**DOI:** 10.1038/s42003-021-02464-9

**Published:** 2021-09-01

**Authors:** Nikita A. Khlystov, Yasuo Yoshikuni, Samuel Deutsch, Elizabeth S. Sattely

**Affiliations:** 1 Department of Chemical Engineering, Stanford University, Stanford, CA USA; 2 U.S. Department of Energy Joint Genome Institute, Lawrence Berkeley National Laboratory, Berkeley, CA USA; 3 Howard Hughes Medical Institute, Stanford University, Stanford, CA USA

**Keywords:** Plant biotechnology, Biocatalysis, Crop waste, Expression systems

## Abstract

Lignin has significant potential as an abundant and renewable source for commodity chemicals yet remains vastly underutilized. Efforts towards engineering a biochemical route to the valorization of lignin are currently limited by the lack of a suitable heterologous host for the production of lignin-degrading enzymes. Here, we show that expression of fungal genes in *Nicotiana benthamiana* enables production of members from seven major classes of enzymes associated with lignin degradation (23 of 35 tested) in soluble form for direct use in lignin activity assays. We combinatorially characterized a subset of these enzymes in the context of model lignin dimer oxidation, revealing that fine-tuned coupling of peroxide-generators to peroxidases results in more extensive C-C bond cleavage compared to direct addition of peroxide. Comparison of peroxidase isoform activity revealed that the extent of C-C bond cleavage depends on peroxidase identity, suggesting that peroxidases are individually specialized in the context of lignin oxidation. We anticipate the use of *N. benthamiana* as a platform to rapidly produce a diverse array of fungal lignin-degrading enzymes will facilitate a better understanding of their concerted role in nature and unlock their potential for lignin valorization, including within the plant host itself.

## Abstract (web summary)

Khlystov et al. introduce a new plant-based system (*N. benthamiana*) as a platform to rapidly produce a diverse array of recombinant fungal lignin-degrading enzymes. This study provides an initial framework to better understand the coordinated efforts of various lignin-degrading enzymes during fungal lignin deconstruction in nature.

## Introduction

The utilization of lignin as a highly abundant renewable feedstock for platform commodity chemicals remains a long-sought goal for over four decades^1^. 1.3 billion tons of lignocellulosic biomass are available each year in the U.S. alone, of which lignin comprises up to 40% by weight^1^. The lack of an established route to lignin deconstruction means that the primary value of industrial lignin by-products is heat and electricity by incineration. Molecular decomposition of lignin would yield monomeric aromatic derivatives with economic value in chemical and material applications typically reliant on petroleum, with vanillin^2^ and phenolic resins^3^ as prominent examples.

Biological catabolism of lignin has been widespread in nature for at least 200 million years^4^, presenting scalable means of its deconstruction through renewable enzymatic catalysis under mild conditions. Wood-decaying fungi are particularly proficient at lignin catabolism and are regarded as the primary agents of lignin biodegradation in nature^5^. Some, such as the model white-rot basidiomycete *Phanerochaete chrysosporium*, have been reported to be capable of degrading 1 g lignin per g mycelium per day^6^. Genomic sequencing of this model species as well as other wood-decaying relatives has uncovered a diverse assortment of genes involved in lignin decomposition^7,8^, making basidiomycetes attractive potential sources of wood-decaying enzymes for biotechnological applications. However, basidiomycetes remain genetically intractable and challenging to cultivate rapidly, rendering their utility in the industrial production of lignin-degrading enzymes unrealized.

Identification and efficient heterologous production of basidiomycete lignin-degrading enzymes for rapid biochemical testing have also proven elusive^9,10^. Lignin biodegradation in nature is largely driven by the oxidative action of heme peroxidases secreted by white-rot basidiomycetes^4,11^. Heme cofactor incorporation, disulfide bond formation, and glycosylation make this class of enzymes particularly challenging to overexpress heterologously^10,12,13^. Model bacterial expression platforms such as *Escherichia coli* are poorly suited for the required post-translational features of these enzymes^14^. Previous characterization of this class of enzymes has relied primarily on in vitro refolding from bacterial inclusion bodies, an inherently time-consuming and inefficient process^9^. Therefore, the development of an expression host comprehensively capable of heterologous production of lignin-degrading enzymes is of paramount importance in establishing a biochemical route to lignin valorization^9^.

The yeast *Saccharomyces cerevisiae* presents a logical choice of model eukaryotic microbial host for the heterologous production of lignin-degrading enzymes. An extensively characterized organism with an abundance of tools available for its genetic manipulation, we reasoned that this fungal relative could readily be engineered for production of basidiomycete lignin-degrading enzymes. However, one potential challenge is that yeast do not natively secrete any heme peroxidases^15^. To this end, we hypothesized that a plant-based expression system could serve as a good candidate chassis for the heterologous production of lignin-degrading heme peroxidases. Plants produce numerous extracellular heme peroxidases for cell wall biosynthesis and morphogenesis, with the widely used experimental host *Nicotiana benthamiana*, a close relative of tobacco, natively producing six class III peroxidases^16,17^. Because of its amenability to protein overexpression through well-established *Agrobacterium* infiltration methods for DNA delivery^18^ and its native capacity to secrete heme peroxidases, we reasoned that *N. benthamiana* would be well-suited as a heterologous host for the production of fungal lignin-degrading peroxidases, and sought to compare its performance to that of *S. cerevisiae* across the major classes of lignin-degrading enzymes.

**Fig. 1:**
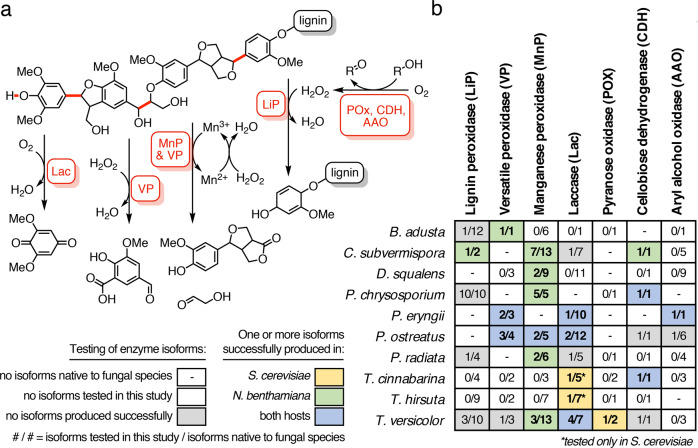
Overview of fungal enzymatic lignin deconstruction in nature. **a** A representative schematic of bond cleavage catalyzed by different families of ligninases. Featured in red are carbon–carbon bonds that can be cleaved by different types of heme peroxidases (MnP, VP, LiP), as well as phenolic bonds that can be cleaved by laccases (Lac). Auxiliary enzymes such as aryl alcohol oxidase (AAO), cellobiose dehydrogenase (CDH), and pyranose oxidase (POX) are shown as examples that can be coupled to peroxidase activity on lignin bonds. **b** An overview of ligninases tested in this study. Each entry indicates the number of isozymes tested out of the total number of isozymes native to each fungal species. Entries bolded and highlighted in yellow indicate successful heterologous production in *S. cerevisiae*, those in green for *N. benthamiana*, and those in blue for both species of one or more isozymes in that category. See Fig. 2 for more detailed activity analysis of individual enzymes. Entries with dashes indicate the absence of that enzyme type in the fungal species, and those having zero as the first number indicate that no representative genes from that species and enzyme class were synthesized or tested. Laccases from *T. cinnabarina* and *T. hirsuta* were tested only in *S. cerevisiae* and are denoted with an asterisk.

Basidiomycetes employ a diverse arsenal of enzymes to achieve lignin degradation (Fig. 1a), featuring numerous isoforms of individual enzyme types^8^. Given their uniquely high redox potential sufficient to perform one-electron oxidation of the aromatic lignin backbone^19^, we prioritized the study of a large array of members from across the three major white-rot peroxidase families. Lignin peroxidases (LiP) are known to oxidize lignin-related compounds at the enzyme surface through long-range electron transfer (LRET), manganese peroxidases (MnP) through oxidation of Mn(III) as a diffusible mediator, and versatile peroxidases (VP) through both modes of oxidation^20^. As these peroxidases use peroxide as a co-substrate, we also selected a number of basidiomycete peroxide-generating enzymes from the pyranose oxidase (POx) and aryl alcohol oxidase (AAO) families to characterize coupled activity alongside the peroxidases. To explore the general capacity of heterologous hosts to produce lignin-degrading enzymes, we also selected members of the laccase (Lac) and cellobiose dehydrogenase (CDH) enzyme families, which are thought to catalyze the oxidation of phenolic lignin fragments and to interface with cellulose deconstruction, respectively^21^.

Individual members of the peroxidase families have been shown to catalyze a reduction in the molecular weight of synthetic lignin in vitro^22,23^; however, many open questions remain surrounding lignin deconstruction in nature. Why do white-rot basidiomycetes possess as many as 26 genes encoding peroxidases^8^, including 13 manganese peroxidase isoforms^24^, and how are their activities coordinated during lignin degradation? While addressing these questions will likely require the development of new genetic tools in fungal lignin-degrading species, we propose that the reconstitution of enzymatic delignification either in vitro or in a heterologous host could also help shed light into biological lignin metabolism. This approach has been limited to date by the inability to rapidly produce and test diverse sets of lignin-degrading enzymes. As a first step toward addressing these broader questions, we sought to develop a general platform for the production of an array of peroxidase isoforms and compare their activity on representative lignin substrates and in combination with other classes of enzymes implicated in fungal lignin degradation. We began by testing the production of an extensive panel of members from the major classes of lignin-degrading enzymes in both the model eukaryotic microbial host *S. cerevisiae* and the model plant *N. benthamiana*. Our results suggest that *N. benthamiana* is better suited for the production of lignin-degrading enzymes and fulfils the role of an effective production platform required for their study, with 23 of 35 enzymes tested expressed in active form. Combinatorial assay of a selected set of successfully produced enzymes for cleavage of a model lignin dimer revealed that the extent of lignin C–C bond scission is enhanced in a coupled system, as well as by the action of specific isozymes compared to others from a given fungal species. Taken together, these results provide insight into the specialization of different isozymes and will help facilitate an effective means of engineering biochemical lignin valorization.

## Results

### Improved heterologous production of lignin-degrading enzymes *in plantae*

**Fig. 2:**
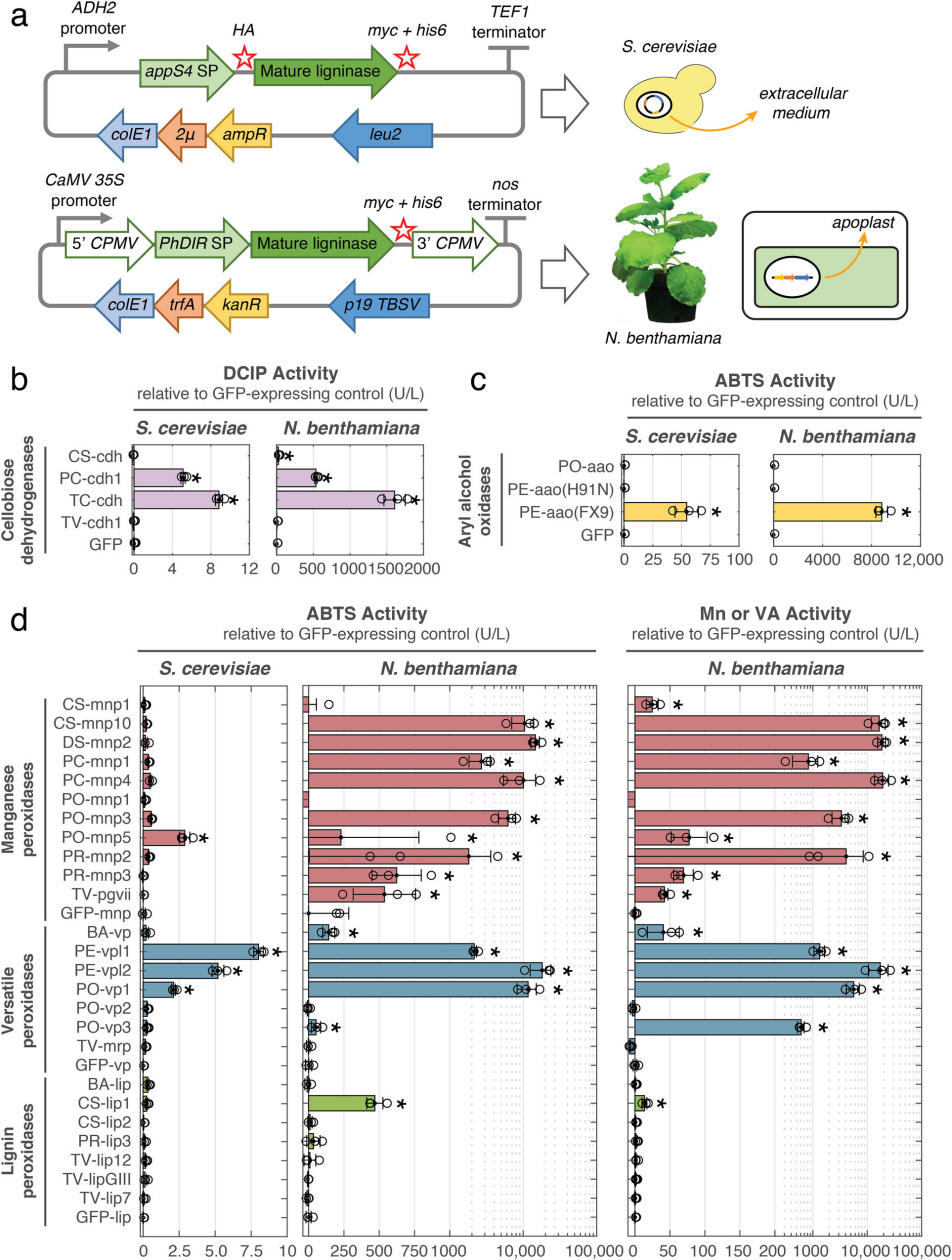
Overview of heterologous production of lignin-degrading enzymes in *S. cerevisiae* and *N. benthamiana*. **a** Genetic components used for heterologous production of lignin-degrading enzymes in *S. cerevisiae* and *N. benthamiana* and enzyme localization after production. For *S. cerevisiae*, production of enzymes was driven by an ADH2 promoter on a pCHINT2AL high-copy expression vector^25^ and exported to the extracellular medium (supernatant) via fusion to an evolved variant of *S. cerevisiae* α-mating-factor signal peptide (*appS4* SP)^48^. Production of enzymes in *N. benthamiana* was driven by a 35S promoter on a pEAQ expression vector^49^ and exported to the plant apoplast via fusion to the signal peptide of dirigent protein from *Sinopodophyllum hexandrum* (*PhDIR* SP). The pEAQ vector includes 5′ and 3′ untranslated regions from cowpea mosaic virus (5′ *CPMV* and 3′ *CPMV*) to enhance expression levels. Hemaaglutinin (*HA*), c-myc (*myc*), and hexahistidine (*his6*) affinity tags were included as N- and/or C-terminal fusions where indicated. **b** Activity of cellobiose dehydrogenases expressed in *S. cerevisiae* and *N. benthamiana*, measured spectroscopically as DCIP reduction. **c** Activity of aryl alcohol oxidases expressed in *S. cerevisiae* and *N. benthamiana*, measured spectroscopically as HRP-coupled oxidation of ABTS in the presence of veratryl alcohol. **d** Summary of lignin-degrading heme peroxidase activity in culture supernatant of *S. cerevisiae* or in apoplast extracts of *N. benthamiana*, assayed against ABTS and veratryl alcohol^38^ or Mn(II)^39^. Lignin peroxidases are shown in green, versatile peroxidases in blue, and manganese peroxidases in red. Mn/veratryl alcohol activity corresponds to Mn(II) activity (1 mM MnSO_4_, 100 µM H_2_O_2_) for manganese and versatile peroxidases and veratryl alcohol activity (20 mM veratryl alcohol, 100 µM H_2_O_2_) for lignin peroxidases. ABTS activity assays (4 mM ABTS, 100 µM H_2_O_2_) of manganese peroxidase samples also included 1 mM MnSO_4_. Activities represent 1 µM oxidized product formed min^−1^ l^−1^. Activities were measured of three different leaves in *N. benthamiana* 5 days after *Agrobacterium* infiltration, or three biological replicates for *S. cerevisiae* after 2 days of cultivation. Reported activities are measured activity levels minus background activity levels in the corresponding GFP-expressing control sample for each set of enzymes (GFP-mnp, GFP-vp, GFP-lip). Data points represent the average of three independent reaction replicates with error bars calculated as one standard deviation. Asterisks indicate statistically significant activity levels relative to GFP control samples (*p* < 0.05, excluding outliers). BA *Bjerkandera adusta*, CS *Ceriporiopsis (Gelatoporia) subvermispora*, PC *Phanerochaete chrysosporium*, PE *Pleurotus eryngii*, PO *Pleurotus ostreatus*, PR *Phlebia radiata*, TC *Trametes (Pycnoporus) cinnabarinus*, TV *Trametes versicolor*.

Our proposed approach to examine the potential synergistic activity of lignin-degrading enzymes (Fig. 1a) requires a comprehensive set of basidiomycete proteins for combinatorial analysis (Fig. 1b). Given the genetic intractability and the native lignolytic background of basidiomycetes, we opted for heterologous production to obtain these enzymes, attempting to do so in *S. cerevisiae* first. Production of enzymes in soluble form without refolding was desirable for rapid screening and required a eukaryotic host to achieve proper folding and post-translational modification of the secreted lignin-degrading enzymes. Sourcing from annotated fungal genomes, we identified 65 genes with previous transcriptomic, secretomic and/or biochemical characterization spanning seven major classes of lignin-degrading enzymes. We codon-optimized and de novo synthesized genes encoding for mature enzymes fused to a variety of yeast-based signal peptides and affinity tags, cloning into low- and high-copy expression cassettes (Fig. 2a). We observed that a strain background repaired of mitochondrial defects^25^ was more proficient in secretion of a model peroxidase compared to the commonly-used secretion strain BJ5465^26^ (Supplementary Fig. 1) and used this strain for heterologous production of our array of fungal genes. We also tested an array of genetic components, media supplements, and growth temperatures (Supplementary Figs. 1 and 2) and arrived at a high-copy expression cassette (Fig. 2a) and cultivation conditions best-suited for high-throughput screening of enzyme production in *S. cerevisiae*.

Using this optimized expression platform, we successfully produced six of 11 laccases, a pyranose oxidase, and two of four cellobiose dehydrogenases using *S. cerevisiae* as indicated by activity assays (Fig. 2b and Supplementary Fig. 3). Aryl alcohol oxidase production was achieved through the use of a previously-evolved, well-expressing enzyme variant^27^ (Fig. 2c). Of greatest interest were the heme peroxidases, since this class of enzymes are considered to be the primary agents of lignin degradation. Out of 47 isozymes, only four were produced to any extent measurable by activity assays toward the model peroxidase substrate 2,2′-azino-*bis*(3-ethylbenzothiazoline-6-sulfonic acid) (ABTS) (Fig. 2d and Supplementary Fig. 4), with no detectable activity toward the lignin-related substrates Mn(II) and veratryl alcohol. We also observed that yeast media supernatant inhibited the activity of commercially available lignin peroxidase toward veratryl alcohol, suggesting interference of secreted yeast metabolites in oxidation of more recalcitrant substrates by lignin-degrading peroxidases (Supplementary Fig. 5).

Given our interest in testing the activity of lignin-degrading peroxidases, we next attempted their production in *N. benthamiana*, which we hypothesized would be more amenable as a heterologous host given its native repertoire of secreted plant peroxidases. In order to test heterologous production of lignin-degrading enzymes in a high-throughput manner, we employed *Agrobacterium*-mediated transient expression of fungal genes in leaf tissue of mature plants. We targeted the proteins to the plant apoplast via C-terminal fusion to the signal peptide of a dirigent protein from *Sinopodophyllum hexandrum* (Fig. 2a) to provide access to post-translational modifications and an appropriate folding environment. This targeting strategy also enabled straightforward extraction of heterologous enzymes in soluble form by centrifugation of leaf tissue^28^ (Supplementary Fig. 6). In contrast to yeast, our approach of using a plant-based host enabled robust heterologous production of a diverse set of isozymes from each of the three major types of white-rot lignin-degrading peroxidases (Fig. 2d). We measured activity levels of raw apoplast extracts of plants expressing fungal peroxidases toward the model peroxidase substrate ABTS and found that members of all three classes of lignin-degrading peroxidases could be successfully produced with levels greater than background activity of corresponding GFP-expressing control extracts (Fig. 2d showing data relative to GFP controls and Supplementary Fig. 7 showing raw data). Raw apoplast extracts also displayed high levels of activity toward veratryl alcohol and Mn(II) substrates without inhibition, in contrast to production in *S. cerevisiae* (Fig. 2d). The differences relative to GFP control were most pronounced in assays involving veratryl alcohol and Mn(II); taken together, these data suggest that 10 manganese peroxidases (MnP), five versatile peroxidases (VP), and a lignin peroxidase (LiP) could be successfully produced in *N. benthamiana*. Further improvements in activity levels were possible through the use of signal peptides native to *N. benthamiana* (Supplementary Fig. 8). *N. benthamiana* also proved proficient in the production of cellobiose dehydrogenases and an aryl alcohol oxidase (Fig. 2b and c). In the case of pyranose oxidase, however, no measurable activity was detected in apoplast extracts from *N. benthamiana*, and *S. cerevisiae* was subsequently used for production of this enzyme (Supplementary Fig. 3). In general, *N. benthamiana* greatly exceeded *S. cerevisiae* in activity of enzyme produced (as determined by comparing activity l^−1^ plant apoplast extract vs. l^−1^ yeast supernatant) and enabled streamlined production of a variety of lignin-degrading enzymes for in vitro lignin activity testing.

### In vitro lignin depolymerization using heterologously produced fungal peroxidases

**Fig. 3:**
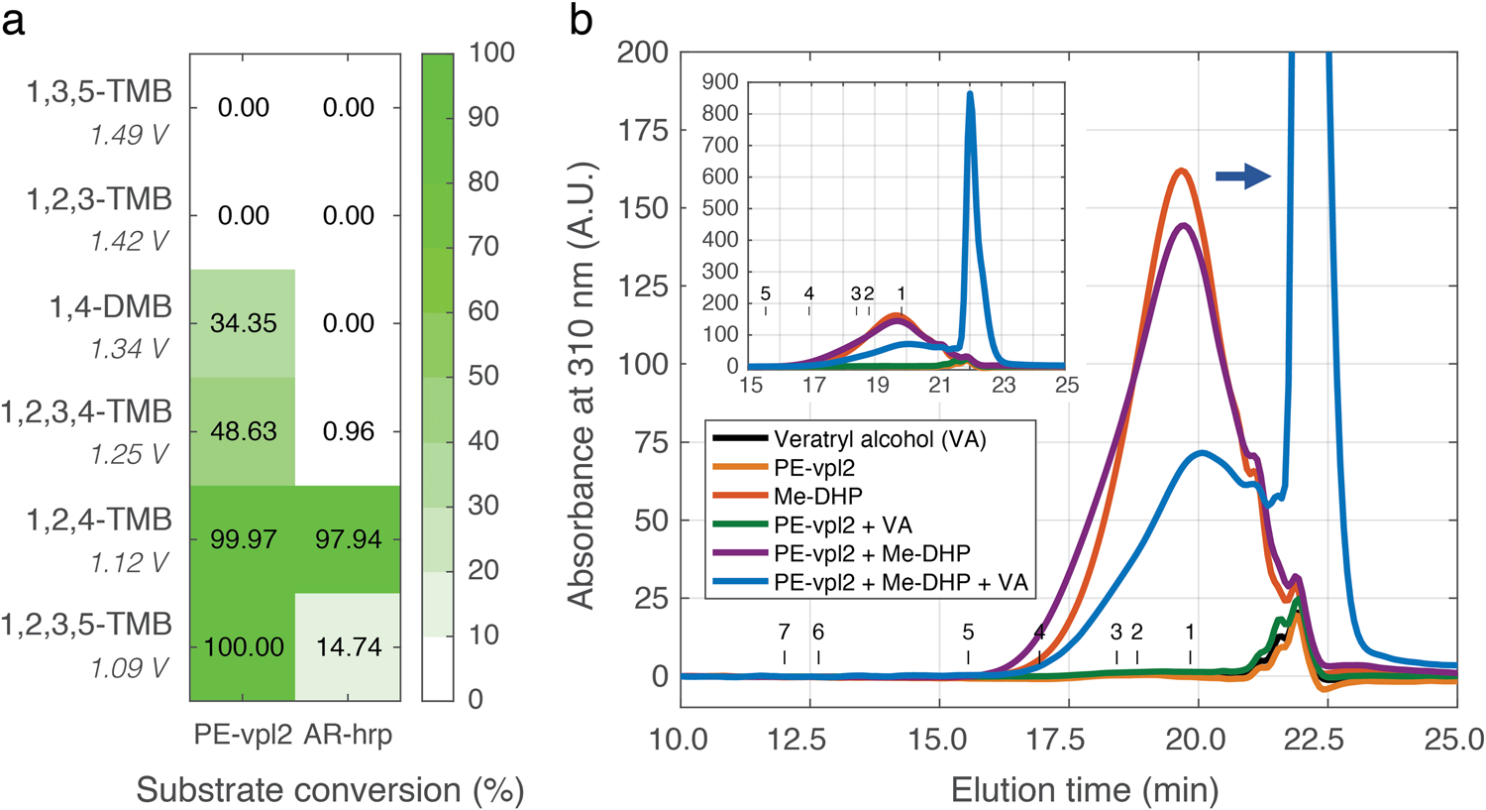
Heterologous lignin-degrading enzymes catalyze in vitro oxidation and depolymerization of lignin-related substrates. **a** Comparison of methoxybenzene oxidation by heterologous FPLC-purified PE-vpl2 extracted from *N. benthamiana* as compared to peroxidase from horseradish. Values represent substrate consumption relative to a no-enzyme control and are the average of three independent reaction replicates. DMB dimethoxybenzene, TMB trimethoxybenzene or tetramethoxybenzene. Redox potentials previously reported^19,50^ for methoxybenzenes are shown for each substrate. **b** Gel permeation chromatography (GPC) of methylated DHP lignin (Me-DHP) subjected to depolymerization by PE-vpl2 purified by FPLC after heterologous production in *N. benthamiana*. Arrows indicate a decrease in lignin molecular weight by PE-vpl2 relative to a no-enzyme control. In vitro conditions involved 200 μg ml^−1^ methylated DHP lignin, 10 mM veratryl alcohol, 0.25% *v/v* Tween-20, and 0.66 μM purified enzyme in 10 mM sodium acetate, pH 4.5. 100 μM hydrogen peroxide was added every 1.5 h for a total of 600 μM. The entire reaction contents were lyophilized and reconstituted in dimethylformamide containing 0.1 M lithium bromide for analysis by GPC. Control reactions had veratryl alcohol omitted (PE-vpl2 + Me-DHP), enzyme omitted (Me-DHP alone), lignin omitted (veratryl alcohol + PE-vpl2), both enzyme and lignin omitted (veratryl alcohol alone), and both lignin and veratryl alcohol omitted (PE-vpl2 alone). Absorbance traces shown here are representative of reaction duplicates. The molecular weights of polystyrene standards used for calibration were as follows: (1) 1.37 kg mol^−1^; (2) 2.93 kg mol^−1^; (3) 4.43 kg mol^−1^; (4) 10.1 kg mol^−1^; (5) 21.7 kg mol^−1^; (6) 139 kg mol^−1^; (7) 281 kg mol^−1^.

Having demonstrated activity toward the model substrates ABTS, veratryl alcohol, and manganese(II), we proceeded to confirm that lignin-degrading peroxidases produced heterologously from *N. benthamiana* also maintained their ability to catalyze oxidation of lignin substrates. We selected PE-vpl2 as a representative lignin-degrading peroxidase and, following purification by anion exchange chromatography, tested its activity toward methoxybenzenes as well as synthetic lignin. Fungal lignin-degrading peroxidases are notable relative to other peroxidases due to their exceptionally high redox potential, enabling them to oxidize recalcitrant substrates such as methoxybenzenes where other peroxidases are unable to^19^. However, little information exists on the redox potentials of versatile peroxidases, unlike lignin and manganese peroxidases. Here, we employed a previously described approach involving methoxybenzene substrates^19^ to compare the activity of the versatile peroxidase vpl2 from *Pleurotus eryngii* purified from *N. benthamiana* to that of the non-lignin-degrading peroxidase from horseradish. We found that the heterologously produced enzyme-catalyzed oxidation of methoxybenzene substrates with redox potentials of up to 1.34 V (previously determined by polarographic voltammetry in non-aqueous conditions) (Fig. 3a). By contrast, under the same reaction conditions and enzyme concentration (as determined by heme absorbance), commercially available horseradish peroxidase oxidized methoxybenzene substrates with redox potential of only up to 1.12 V, consistent with previous reports^19^.

Having shown that heterologously produced versatile peroxidase exhibits the high redox potential characteristic of lignin-degrading peroxidases, we attempted in vitro depolymerization of dehydrogenated polymerizate (DHP) lignin as a model substrate. Using DHP lignin having an average molecular weight of ~1500 g/mol (corresponding to ~5-mers) synthesized from sinapyl and coniferyl monolignols^29^, we found that versatile peroxidase oxidation resulted in lignin with significantly higher molecular weight, as indicated by gel permeation chromatography (GPC) (Supplementary Fig. 9a). We hypothesized that methylation of phenolic functional groups in the lignin polymer would prevent the formation of reactive phenolic radical intermediates and subsequent interchain coupling^30,31^. We tested this hypothesis by comparing versatile peroxidase oxidation of a phenolic model lignin dimer relative to a methylated version of the same dimer. We identified coupling products putatively corresponding to tetramers of the lignin dimer in the case of phenolic dimer but not that of the methylated dimer (Supplementary Fig. 9b). Accordingly, we performed methylation of DHP lignin and found that oxidation by versatile peroxidase PE-vpl2 resulted in a decrease in lignin molecular weight as well as appearance of low molecular weight products, suggestive of in vitro lignin depolymerization (Fig. 3b). Taken together, our results indicate that lignin-degrading peroxidases produced heterologously from *N. benthamiana* retain their unique native catalytic properties toward recalcitrant lignin-like substrates.

### Model lignin dimer oxidation using a panel of lignin-degrading enzymes individually and in combination

Equipped with a diverse set of heterologously produced fungal lignin-degrading enzymes, we sought to uncover their coordinated roles in the context of lignin degradation. The insolubility and random chemical composition of whole lignin posed challenges with regards to quantifying chemical modifications by our set of enzymes in a high-throughput manner. To circumvent this, we selected a β-O-4 dimer as a model representative of the most common type of linkage found in lignin^32^, enabling direct quantification of a given enzyme’s ability to oxidize and/or cleave this representative lignin bond using liquid-chromatography mass spectroscopy (LC-MS). Given the sensitivity of lignin-degrading peroxidases to suicide inactivation by excess peroxide^33^, we also sought to better approximate biological delignification by providing peroxide via an enzyme coupling approach rather than by direct addition of peroxide to help mitigate this issue. This was achieved by coupling peroxidases to peroxide-generating enzymes such as sugar oxidases and aryl alcohol oxidases, thought to be the primary sources of peroxide in wood-decaying basidiomycete fungi^34–37^. We selected a representative from each type of lignin-degrading peroxidase: CS-lip1 as a lignin peroxidase; PC-mnp1 as a manganese peroxidase; and PE-vpl2 as a versatile peroxidase. We chose a pyranose oxidase from *T. versicolor* and produced in *S. cerevisiae*, the evolved variant of aryl alcohol oxidase^27^ from *P. eryngii* and produced in *N. benthamiana*, and commercially available glucose oxidase from *A. niger* as peroxide-supplying counterparts to the peroxidases in the coupled reactions.

**Fig. 4:**
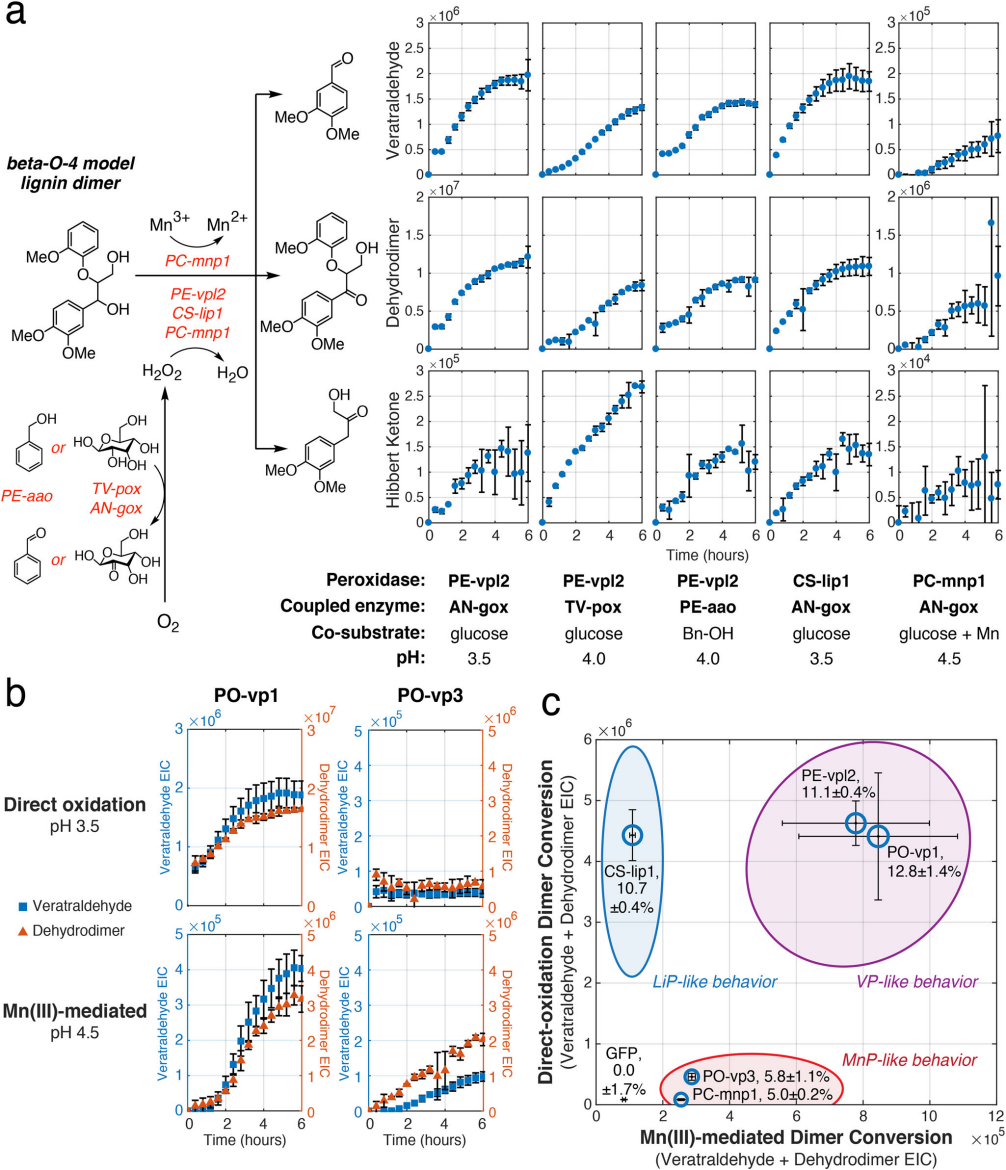
Cleavage of a model β-O-4 lignin dimer using heterologous lignin-degrading enzymes produced by *N. benthamiana*. **a** Diafiltrated extracts of a versatile peroxidase (PE-vpl2), a lignin peroxidase (CS-lip1), or a manganese peroxidase (PC-mnp1) were coupled with peroxide-generating sugar oxidases (TV-pox or AN-gox) or aryl alcohol oxidase (PE-aao(FX9)) in the presence of corresponding co-substrates and under the indicated pH conditions. PE-aao but not PE-vpl2 was found to have activity toward benzyl alcohol (Bn-OH), meaning this coupling reaction could proceed without interference between the two enzymes (Supplementary Fig. 15). Decomposition products (veratraldehyde, dehydrodimer, and Hibbert ketone) were tracked over the course of the reaction using LC-MS. **b** Dimer oxidation by GOx-coupled diafiltrated extracts of two isoforms of versatile peroxidase from *P. ostreatus* (PO-vp1 and PO-vp3) were compared to PE-vpl2, CS-lip1, and PC-mnp1 under direct oxidation and Mn(III)-mediated oxidation conditions. **c** Activity phase diagram of white-rot lignin-degrading peroxidases. The extent of dimer conversion to veratraldehyde and dehydrodimer in reactions containing peroxidase isozymes coupled to glucose oxidase was measured under direct-oxidation conditions (pH 3.5) and Mn(III)-mediated conditions (pH 4.5 with 1 mM MnSO_4_). The extent of dimer cleavage was calculated as the proportion of veratraldehyde EIC relative to the sum of veratraldehyde EIC and dehydrodimer EIC. The maximum dimer cleavage extent under either condition is displayed in the peroxidase isozyme labels and represented by marker size. Data points represent the average of three independent reaction replicates with error bars calculated as one standard deviation.

Through kinetic sampling of the reactions by LC-MS, we identified three major products resulting from the oxidation of the model dimer^32^: veratraldehyde and a Hibbert ketone, resulting from carbon–carbon bond cleavage; and dehydrodimer, resulting from oxidation of the α-hydroxy group without bond cleavage (Fig. 4a). We observed accumulation of these products in all coupled reactions containing diafiltrated extracts of lignin-degrading peroxidases. Although we observed significant background ABTS oxidation activity in GFP-expressing control extracts, no dimer oxidation was observed in the case of GFP-expressing extracts or in the absence of a peroxide-generating enzyme, indicating that dimer conversion was specific to the combined activity of heterologously produced lignin-degrading enzymes and not other proteins native to the *N. benthamiana* apoplast extract (Supplementary Figs. 10 and 11). Accumulation of guaiacol as the second product resulting from dimer cleavage was not observed, likely due to its subsequent polymerization by the peroxidase.

Fungal lignin-degrading heme peroxidases have previously been demonstrated to catalyze lignin depolymerization through direct interaction with lignin substrates at the enzyme surface facilitated by long-range electron transfer (direct-oxidation mode)^38^ and/or through Mn(III) as a diffusible mediator that transfers electrons from lignin to the enzyme active site (indirect oxidation)^39^. Lignin peroxidases, having the necessary surface-exposed tryptophan residue, have been shown to operate by direct oxidation, while manganese peroxidases, lacking the tryptophan residue and instead having a Mn(II) binding site, operate by indirect oxidation; versatile peroxidases are able to function via both modes of oxidation depending on reaction conditions. The presence of Mn(II) and pH 4–5 promotes indirect oxidation, while pH < 3.5 and the absence of Mn(II) limits the possible modes of reaction to direct oxidation. We sought to better understand which of the two modes of oxidation is more effective in cleavage of the common β-O-4 lignin linkage. We tested the activity of heterologously produced peroxidases toward the lignin model dimer in both modes of oxidation and found that total product formation occurred to a greater extent under conditions favoring direct substrate oxidation at the enzyme surface as compared to indirect substrate oxidation via Mn(III) (see PE-vpl2 & CS-lip1 at pH 3.5 & 4.0 without Mn(II), vs. PC-mnp1 & PE-vpl2 at pH 4.5 with the inclusion of Mn(II) and malonic acid as a chelator, respectively) (Fig. 4a and Supplementary Fig. 12). Of the different observed products, C–C bond cleavage was also more limited in the case of MnP-like enzymes (e.g., PC-mnp1) compared to the other two peroxidase classes, as indicated by the proportion of veratraldehyde detected relative to dehydrodimer (Supplementary Fig. 12). In comparing the activity of peroxidases without added Mn(II), products accumulated to a similar extent regardless of the enzyme used to generate peroxide in situ (Fig. 4a, PE-vpl2 & CS-lip1 with AN-gox, TV-pox, or PE-aao).

Our collection of enzymes successfully produced from *N. benthamiana* included two isoforms of versatile peroxidase from *P. ostreatus*, PO-vp1, and PO-vp3. This provided an opportunity to investigate the specialized roles of the numerous isozymes involved in fungal lignin degradation by analyzing their capacity for model dimer oxidation under different conditions. We observed that coupled reactions involving PO-vp1 produced substantially greater conversion of lignin dimer compared to PO-vp3, especially under direct-oxidation conditions (Fig. 4b). Comparing the maximal dimer cleavage extent across oxidation conditions, we found that C–C bond cleavage by PO-vp1 also exceeded that of PO-vp3 (Fig. 4c and Supplementary Fig. 12). Together, these findings suggest that PO-vp1 is better tailored for oxidation as well as cleavage of this type of lignin bond compared to its relative PO-vp3 in any oxidation mode.

We expanded this approach to all isozymes tested in the coupled context. Our technique involving high-throughput LC-MS reaction analysis paired with rapid soluble production of multiple isozymes through the *N. benthamiana* platform enabled us to construct a quantitative activity phase diagram portraying the characteristic behavior of isozymes on the model lignin dimer substrate (Fig. 4c). We constructed this diagram using dimer conversion extents of each of the isozymes under direct and Mn(III)-mediated oxidation conditions, and identified three behavioral regimes of dimer oxidation, consistent with the three identified families of white-rot lignin-degrading peroxidases. However, unlike previous categorization of isozymes based on activity toward substrates that do not model lignin linkages, such as veratryl alcohol and Mn(II)^9^, this diagram reflects their specific behavior on a major lignin bond type, suggesting that isozymes such as PO-vp3 exhibit MnP-like interactions with lignin despite its classification as a versatile peroxidase.

### Coupling condition optimization

In an attempt to inform engineering efforts aimed at lignin deconstruction, we sought to identify reaction conditions that maximized conversion of lignin dimer. Given that our results indicated that coupling was an advantageous approach to dimer oxidation (Fig. 4), we attempted to further optimize coupling conditions to achieve maximal dimer conversion and to also better understand peroxidase sensitivity to suicide inactivation by excess peroxide in this context. Our results indicated that model dimer conversion occurred most effectively under conditions favoring direct oxidation (Fig. 4c), irrespective of the peroxide-generating enzyme used (Fig. 4a). Accordingly, we selected the coupling of FPLC-purified PE-vpl2 and commercially-purified AN-gox (Sigma) for optimization and focused on the peroxide generation rate as a function of AN-gox concentration.

**Fig. 5:**
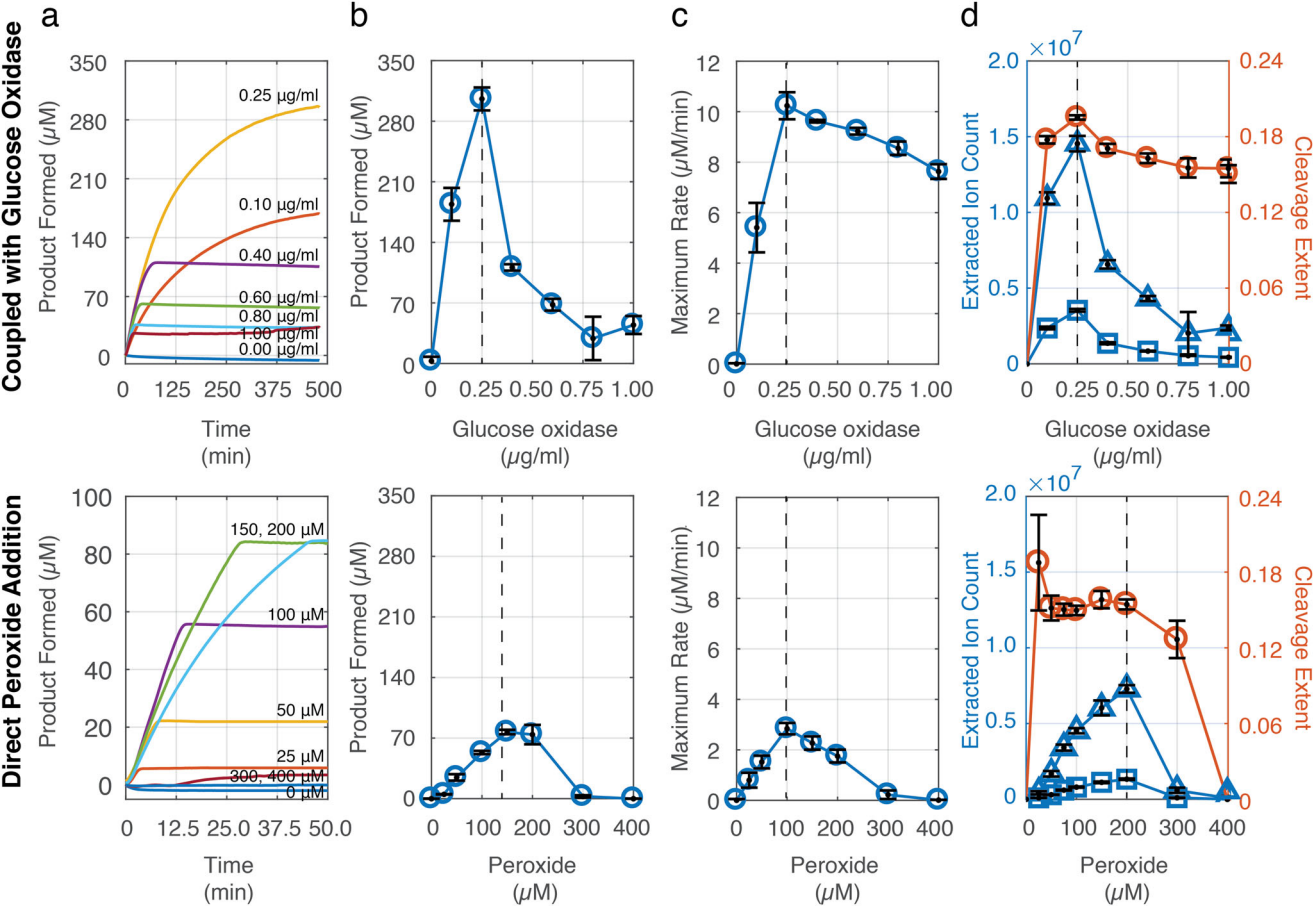
Coupling to in situ peroxide production by glucose oxidase enables greater conversion of a model lignin dimer by the lignin-degrading peroxidase PE-vpl2. **a** Kinetic traces of dimer oxidation as a function of glucose oxidase concentration (top) and exogenously added peroxide (bottom). Absorbance was measured at 310 nm and converted to an estimated micromolar aldehyde produced using the molar extinction coefficient for veratraldehyde (9300 M^−1^ cm^−1^). Kinetic reactions were performed in triplicate, with a representative time trace shown here. **b** Maximal dimer conversion achieved over the course of the assay as a function of glucose oxidase concentration (top) or peroxide (bottom), using the molar extinction coefficient for veratraldehyde. **c** Maximum oxidation rate observed during dimer oxidation as a function of glucose oxidase concentration (top) or peroxide (bottom). **d** LC-MS analysis of the reaction products produced by dimer oxidation as a function of glucose oxidase concentration (top) or peroxide (bottom). The extracted ion counts (EIC), normalized to the conditions lacking glucose oxidase or peroxide, are shown in blue (triangles, dehydrodimer; squares, veratraldehyde). The cleavage extent (red) was calculated as the ratio of veratraldehyde EIC to the sum of dehydrodimer and veratraldehyde EIC. All reactions were performed at 25 °C and pH 4.0 containing 0.2 μM versatile peroxidase vpl2 from *P. eryngii* produced and FPLC-purified from apoplast extracts of transgenic *N. benthamiana*. Data points represent the average of three independent reaction replicates with error bars calculated as one standard deviation.

For a given amount of PE-vpl2, coupling to glucose oxidase was a more effective strategy for achieving dimer conversion than direct addition of peroxide, enabling approximately fourfold greater product accumulation (Fig. 5a, b). In the coupled enzyme reaction, the total product formation seems to be limited only by the stability of glucose oxidase under the conditions of the assay (Supplementary Fig. 13). Coupling successfully overcame the limit on conversion in the case of direct peroxide addition, where increasing peroxide concentration did not result in additional product formation, likely due to peroxidase inactivation (Fig. 5a, b, bottom). We identified an optimum glucose oxidase concentration, below which the rate of the coupled reaction limited dimer conversion within the lifetime of glucose oxidase, and beyond which peroxide likely was generated in excess and resulted in peroxidase inactivation as evidenced by rapid loss of activity (Fig. 5a, b, top). This maximum concentration could be extended through addition of catalase as well as increasing the concentration of peroxidase to accelerate the rate of reaction and therefore enable greater substrate conversion within the lifetime of glucose oxidase (Supplementary Fig. 13). Given the importance of maximizing lignin bond breakage in the process of lignin degradation, we also compared the extent of dimer C–C bond cleavage in the two methods of peroxide addition (Fig. 5d). We found that at the optimal glucose oxidase concentration, coupling significantly enhanced bond scission compared to the direct addition of peroxide at any concentration, indicating that our coupling approach would be desirable in engineering synthetic routes to lignin deconstruction.

## Discussion

Through the use of *Agrobacterium-*mediated transient expression in *N. benthamiana*, we achieved soluble heterologous production of the majority of basidiomycete enzymes tested (23 of 35). This represents a significant step in overcoming a major obstacle in the study of this class of enzymes and enables rapid combinatorial characterization of their concerted roles in lignin biodegradation. Coupled with LC-MS kinetic analysis, our approach afforded new and technologically valuable insights into the activity of these enzymes in the context of the most commonly occurring linkage in lignin. Whereas previously limitations in the production of lignin-degrading enzymes meant that they were characterized individually, in this study we were able to quantitatively compare the capacity of a set of lignin-degrading peroxidase isoforms to oxidize and cleave this ubiquitous lignin bond under different oxidation modes.

We found that coupling to a peroxide-generating enzyme such as a sugar oxidase enabled greater peroxidase-catalyzed dimer oxidation as well as bond cleavage compared to direct addition of peroxide, suggesting that coupling would be desirable in the context of enzymatic valorization of lignin, especially with peroxide-generating enzymes optimized for greater stability under assay conditions. Our data (Fig. 5) corroborates prior work that excess peroxide is detrimental to peroxidase activity, suggesting that fungi are able to tightly control the provision of peroxide during lignin breakdown in order to avoid peroxidase inactivation and maintain efficient lignin depolymerization. One possible method by which fungi could achieve this is through co-localization of enzymes into an extracellular metabolon or other pseudo-organized structures akin to cellulosomes^40^, although no data has been collected to date to support this hypothesis. In this coupled context, we also discovered that certain isoforms within a peroxidase family are significantly more proficient in the oxidation and cleavage of this model lignin dimer than others. In the context of natural lignin biodegradation, this suggests some isoforms are more specialized for the initial stages of lignin oxidation, while the activity of other isoforms toward simpler substrates such as Mn(II) and dimethoxyphenol^23^ indicates they may be more involved in downstream oxidation of lignin degradation products.

As seen in this study, enzyme categorization based on small-molecule proxies such as ABTS, veratryl alcohol, or Mn(II) does not adequately predict isozyme behavior toward lignin bonds. Further activity testing toward an augmented set of substrates, including those that better reflect the insolubility and crosslinked properties of lignin within natural lignocellulosic biomass, is required for a complete mechanistic understanding. The coupled kinetic characterization approach described here could be readily applied for the study of isozyme specialization toward other commonly occurring lignin linkages such as 5-5′ and β-5 through suitable synthetic model dimers. Activity measurements toward synthetic trimers^41^ and oligomers would further elucidate substrate affinities amongst the large collection of enzyme isoforms involved in fungal lignin degradation. For instance, using the 10 manganese peroxidases successfully produced using our heterologous platform, lignin-specific substrate preferences could be revealed for this seemingly redundant collection of manganese-oxidizing isoforms. While we found that PO-vp3 is inactive toward the β-O-4 lignin linkage under direct-oxidation conditions (pH 3.5, no Mn(II)), it may have yet-to-be-discovered activity toward other components of the lignin backbone. Using two-dimensional nuclear magnetic resonance (NMR)^42^, the gap between activity characterization based on synthetic small-molecule lignin bond representatives and that based on whole lignin can be bridged to further reveal mechanistic insights on the chemical modifications imparted by each different peroxidase isoform. Combined with our findings described here regarding the β-O-4 dimer, this information about isozyme specialization could serve as initial guidelines for engineering a biochemical route to lignin deconstruction.

The substantially increased capacity of *N. benthamiana* relative to *S. cerevisiae* for the heterologous production of a variety of fungal lignin-degrading enzymes highlights the challenges of selecting an appropriate chassis for these enzymes, especially the heme peroxidases. For example, we observed numerous high molecular weight bands on Western blotting of lignin-degrading peroxidases in *S. cerevisiae* (Supplementary Fig. 14) compared to single-defined glycoforms of *N. benthamiana* apoplast extracts (Supplementary Fig. 10), suggesting hyperglycosylation in the yeast host. This is in line with previous reports suggesting mannosylated proteins tend to suffer from hyperglycosylation in yeast^13^, an indication of misprocessing of the nascent heterologous protein in the ill-equipped yeast organism. Auxiliary activities by chaperone proteins may also be necessary for full maturation of lignin-degrading enzymes in native fungal species, as previous research has shown that mannose structures of lignin peroxidase from *P. chrysosporium* are post-translationally dephosphorylated by an extracellular enzyme^43^ and that propeptide processing may be responsible for proper formation of functional tetrameric complexes of pyranose oxidase from the same organism^44^. Adequate supply of cofactors such as heme can also expected to be essential for functional overproduction of lignin-degrading enzymes, particularly peroxidases. We observed robust heterologous production of lignin-degrading heme peroxidases using our plant-based platform, especially in comparison to yeast. This suggests that *N. benthamiana* has sufficient supply of heme to the secretory pathway, presumably due to native production of peroxidases and laccases involved in plant cell wall biogenesis. The larger panel of heterologous enzymes may also be possible due to the fact that *N. benthamiana* natively handles a wider range of proteins targeted for the secretory pathway compared to *S. cerevisiae*, especially for the case of heme-containing proteins^15,17^. These factors underscore the benefit of utilizing the model plant host’s well-suited native secretory machinery to produce heterologous secreted heme peroxidases.

As synthetic biology for the study of basidiomycete genetics remains in its infancy, the cellular components necessary for successful folding and processing of lignin-degrading enzymes remain unknown. The results shown here in *S. cerevisiae* as well as previous studies in filamentous fungi^10^ suggest that these simpler, genetically tractable organisms lack the specialized cellular environments required for functional expression of lignin-degrading enzymes. The extraordinary oxidative potential of the heme peroxidases in particular raises questions regarding possible specialized mechanisms of basidiomycete fungi and presumably plant species to handle the oxidative stress resulting from the production of these peroxidases. In particular, the relative ease of heterologous production of different manganese peroxidases compared to lignin peroxidases in *N. benthamiana* (Fig. 2d) suggests protein-level determinants of successful folding and export. The development of *N. benthamiana* as a platform amenable to genetic engineering and proficient at lignin-degrading enzyme production affords the opportunity to develop hypotheses aimed at uncovering key cellular components for each class of enzyme, which could inform tailoring of other heterologous production platforms for large-scale production of these biotechnologically important enzymes.

The expression of basidiomycete lignin-degrading enzymes in a lignified plant host also enables *in planta* studies focused on biomass valorization. The transient expression platform described here could be harnessed to trigger lignin decomposition at late stages of plant growth, for instance to facilitate improved biosaccharification by increasing enzyme accessibility of cellulose, augmenting previous work involving bacterial lignin-degrading enzymes^45,46^. This inducible strategy is advantageous in that plant fitness effects would be mitigated relative to existing strategies^47^ aimed at reducing and/or modifying lignin biosynthesis. Through metabolomic analysis and lignin visualization techniques, our platform also provides a framework for characterization of heterologous lignin-modifying enzymes in the context of the plant host itself. Analogous to our in vitro approach described here, combinatorial expression of these enzymes in planta would provide insight into their roles within the more complex and therefore more informative environment of plant tissue. The ever-evolving array of synthetic biology tools available for plant hosts would further help augment our understanding of enzymatic lignin depolymerization. We anticipate that both in vitro and *in planta* analysis of enzymatic delignification will help advance biorefinery utilization of the millions of tons of lignocellulose available globally each year.

## Methods

### Chemicals and reagents

Veratryl alcohol (3,4-dimethoxybenzyl alcohol, A13396) and ABTS (2,2′-azino-*bis*[3-ethylbenzothiazoline-6-sulfonic acid] diammonium salt, J65535) were purchased from Alfa Aesar (Haverhill, MA, USA). β-O-4 dimer (1-[3,4,-dimethoxyphenyl]-2-[2-methoxyphenoxy]propane-1,3-diol, AK-40175) was purchased from Ark Pharm (Arlington Heights, IL, USA). DCIP (dichloroindophenol, D1878) was purchased from Sigma-Aldrich (St. Louis, MO, USA).

Glucose oxidase from *Aspergillus niger* (G2133) and lignin peroxidase from *Phanerochaete chrysosporium* (42603) were purchased from Sigma-Aldrich (St. Louis, MO, USA). Peroxidase from horseradish (31941) was obtained from SERVA (Heidelberg, Germany).

### Protein expression in *S. cerevisiae*

*S. cerevisiae* strain JHY693^25^ (gift of J. Horecka and A. Chu, Stanford Genomic Technology Center) was used as the background strain for all yeast protein expression. Ligninase genes were codon-optimized (Gen9) for expression in *S. cerevisiae* and synthesized de novo using DNA sequences coding for the mature enzymes sourced from UniProt and/or MycoCosm (Joint Genome Institute) databases. For single-copy expression vectors, a pRS415-based cassette (gift of C. Harvey, Stanford Genomic Technology Center) was used; for multi-copy expression vectors, the 2-micron cassette pCHINT2AL^25^ was used (gift of C. Harvey). Inducible expression was driven by the ADH2 promoter of *S. cerevisiae*, and ER targeting and secretion of ligninases were achieved by N-terminal fusion to an evolved variant of the α-mating-factor prepropeptide of *S. cerevisiae*^48^. Yeast transformation was carried out using the Frozen-EZ Yeast Transformation II Kit (Zymo Research). Transformant selection was performed using synthetic defined media plates deficient in leucine. Single colonies were picked into 0.5 ml SD-leu media in a 96-well culture plate and incubated overnight with orbital shaking (425 rpm, 30 °C). After centrifugation (600 × *g*, 10 min), the supernatant was removed, and the cell pellets resuspended in supplemented YPEG media (2% ethanol, 3% glycerol, 70 mM potassium phosphate pH 6.0; for heme peroxidase production, 0.01 mM hemin + 1 mM CaCl_2_; for cellobiose dehydrogenase production, 0.01 mM hemin; for laccase production, 2 mM CuSO_4_; no additional supplements for pyranose oxidase or aryl alcohol oxidase production) and incubated for 48 h with orbital shaking (425 rpm, 20 °C). After centrifugation (600 × *g*, 10 min), the culture supernatant was used for subsequent activity assays at 10% v/v.

### Protein expression in *N. benthamiana*

*Agrobacterium*-mediated transient expression was performed as described before^51^. Genes encoding mature ligninases were cloned from *S. cerevisiae* vectors above into the pEAQ-HT expression cassette^49^. The signal peptide of the dirigent protein of *Sinopodophyllum hexandrum* (UniProt A0A059XIK7, residues 1–27) was used to direct protein export to the apoplast. Expression of ligninases was driven by a 35S promoter from cauliflower mosaic virus (CaMV) and a 5′UTR from cowpea mosaic virus (CPMV). *Agrobacterium* strains harboring the expression cassette and a p19-silencing plasmid were grown on dual-selecting kanamycin-gentamycin LB plates (30 °C, 2 days), incubated in induction buffer (150 μM acetosyringone, 10 mM MgCl_2_, 10 mM sodium succinate, pH 5.6; 4–5 h) before being infiltrated into the three youngest leaves of 5- to 7-week-old *N. benthamiana* plants at an OD_600_ of 0.3 in induction buffer. Four days post-infiltration, apoplastic contents were extracted as previously described with modifications^28^. Leaves were harvested and submerged in ice-cold extraction buffer (0.1 M sodium acetate, 0.3 M NaCl, pH 4.5); it was observed that 2-(N-morpholino)ethanesulfonic acid (MES) buffer has an inhibitory effect on peroxidase activity (Supplementary Fig. 16). Leaves were subjected to vacuum cycles (>650 mmHg, 3 × 5 min) to infiltrate the leaves with buffer. Leaves were individually placed on a piece of Parafilm and rolled around a pipette tip. This assembly was inserted into a 5-ml plunger-less syringe contained in a 15-ml conical centrifuge tube and centrifuged (1600 × *g*, 10 min, 4 °C) to produce the apoplastic extract, which was further clarified via centrifugation to remove any plant debris (21,000 × *g*, 10 min, 4 °C). In experiments featuring diafiltrated apoplast extracts, extracts were diafiltrated at least 600-fold with 20 mM sodium tartrate, pH 4.5, 10% *v/v* glycerol through Amicon Ultra-4 10-kDa MWCO centrifugal filters units (EMD Millipore). For sample storage, 1 volume of 40% glycerol was added to 3 volumes of extract and stored at −80 °C.

### Enzyme activity testing

ABTS activity assays were performed using 4 mM ABTS, 100 μM H_2_O_2_, 50 mM sodium tartrate, pH 3.5. Assays for Mn-dependent ABTS oxidation were performed using 4 mM ABTS, 100 μM H_2_O_2_, 1.0 mM MnSO_4_, and 50 mM sodium malonate, pH 4.5. ABTS oxidation kinetics were observed at 414 nm (extinction coefficient 36,000 1/M 1/cm) using a Synergy HTX plate reader at 25 °C. Veratryl alcohol activity^38^ was measured as the production of veratraldehyde at 310 nm (extinction coefficient 9300 1/M 1/cm) using 20 mM veratryl alcohol, 100 μM H_2_O_2_, 50 mM sodium tartrate, pH 3.5, at 25 °C. Manganese-dependent activity^39^ was measured by Mn(III)-malonate complex formation using 1.0 mM MnSO_4_ and 100 μM H_2_O_2_ in 50 mM sodium malonate (270 nm, 11,590 1/M 1/cm) at 25 °C. Cellobiose dehydrogenase activity was measured at 522 nm using 10% *w/v* cellobiose, 0.3 mM dichloroindophenol, and 50 mM sodium tartrate, pH 5.0, at 25 °C. Pyranose oxidase activity was measured by coupling to ABTS as above with the inclusion of 1 μg commercial horseradish peroxidase (HRP) and 2% *w/v* D-glucose in 50 mM sodium acetate, pH 6.0. For all assays, 1 unit of activity is defined as 1 μmol of observable product per liter per minute, and activities are calculated as the maximum observed rate during the initial phase of the enzyme assays.

### LC-MS kinetic analysis of dimer oxidation

All reactions contained 20 mM β-O-4 dimer and peroxidase-containing diafiltrated extract from *N. benthamiana* to 0.2 µM total heme content as determined by the pyridine hemochromagen assay^52^. Glucose oxidase assays contained 0.4% *w/v* D-glucose and either 1.0 ng/μl glucose oxidase and 50 mM sodium tartrate pH 3.5, or 0.574 ng/μl glucose oxidase and 50 mM sodium malonate pH 4.5 with 1.0 mM MnSO_4_, where the glucose oxidase concentration was adjusted between the two pH conditions to keep the rate of peroxide generation constant. Aryl alcohol oxidase assays contained 10 mM benzyl alcohol, 40 U/L (HRP-coupled ABTS activity) of diafiltrated extract of PE-aao(FX9) from *N. benthamiana*, and 50 mM sodium tartrate pH 4.0. Pyranose oxidase assays contained 0.4% *w/v* D-glucose, 10 U/L (HRP-coupled ABTS activity) of diafiltrated supernatant of TV-pox from *S. cerevisiae*, and 50 mM sodium tartrate pH 4.0. Reactions were clarified (21,000 × *g*, 5 min) and initiated by the addition of peroxide-generating enzyme.

Model lignin dimer LC-MS kinetic assays were performed using an Agilent 6545 UHPLC Q-TOF running in positive mode with a 6-min water-acetonitrile gradient (A: water + 0.1% formic acid, B: acetonitrile + 0.1% formic acid: 0 min, 95% A; 0.2 min, 95% A; 3.65 min, 37.5% A; 3.66 min, 5% A; 4.11 min, 5% A; 4.15 min, 95% A; 5.18 min, 95% A; flow rate 0.8 ml/min) on an Agilent RRHD EclipsePlus 95 Å C18 column (2.1 × 50 mm, 1.8 µm, 1200 bar). Reaction product profiles were measured every 24 min by 1 µl direct injection of reaction vials, which were maintained at 22 °C in the autosampler. Extracted ion counts (EIC) were obtained using the ‘Find by Formula’ function in Agilent MassHunter Qualitative Analysis software, using 35 ppm mass tolerance, 35, 500, and 35 ppm symmetric expansion of values for chromatogram extraction for C_9_H_10_O_3_ (veratraldehyde), C_18_H_20_O_6_ (dehydrodimer), and C_11_H_14_O_4_ (Hibbert ketone), respectively. Possible charge carriers and neutral losses were specified as -e^−^, +H, +Na, +K, +NH_4_, and −H_2_O.

### LC-MS analysis of dimer cleavage extent by peroxidase isozymes

Reactions contained 20 mM β-O-4 dimer and 0.4% *w/v* D-glucose. Reactions assaying direct substrate oxidation contained 50 mM sodium tartrate, pH 3.5, and 1.0 ng/μl glucose oxidase; reactions assaying Mn-mediated substrate oxidation contained 50 mM sodium malonate, pH 4.5, 1 mM MnSO_4_, and 0.574 ng/μl glucose oxidase (adjusted to keep reaction rate similar). The amounts of diafiltrated extracts of PO-vp1 and PO-vp3 used in the reactions were normalized to the Mn(II) activity of PC-mnp1 at a reaction concentration of 0.2 µM (total heme content; ~6 U/L). The amounts of diafiltrated extracts of PE-vpl2 and CS-lip1 used in the reaction were normalized to PC-mnp1 by total heme content. Diafiltrated extract of GFP-expressing *N. benthamiana* was used as a negative control at 1% *v/v* (total heme content ~0.07 µM) with the addition of 33.3 ng/μl commercial horseradish peroxidase in order to prevent peroxide accumulation. Reactions were clarified (21,000 × *g*, 5 min) prior to initiation by addition of glucose oxidase or hydrogen peroxide. After 9-h incubation at room temperature, samples were moved to the LC-MS autosampler maintained at 10 °C and analyzed by 1 µl direct injection of the reaction contents on an Agilent 6545 Q-TOF running in positive mode with a 6-min water-acetonitrile gradient (as above) and an Agilent RRHD EclipsePlus 95 Å C18 column (2.1 × 50 mm, 1.8 µm, 1200 bar). EIC values were obtained as above.

### Coupled condition optimization

Reactions contained 20 mM β-O-4 dimer, 50 mM sodium tartrate, pH 4.0, and 330 U/L ABTS activity of FPLC-purified^53^ PE-*vpl2* heterologously produced in *N. benthamiana*. Coupled reactions additionally contained 0.4% *w/v* D-glucose. Absorbance corresponding to the formation of dehydrodimer and veratraldehyde was measured at 310 nm using a Synergy HTX plate reader and converted to an estimate of total aldehyde produced using the molar extinction coefficient for veratraldehyde (9300 1/M 1/cm). Reactions were initiated by the addition of peroxide or glucose oxidase.

After completion, 1 μl of the reaction was injected on a 6545 Agilent UHPLC Q-TOF running in positive mode with an 8-min water-acetonitrile gradient (0 min, 95% A; 0.2 min, 95% A; 5.65 min, 37.5% A; 5.66 min, 5% A; 6.11 min, 5% A; 6.15 min, 95% A; 7.18 min, 95% A; A: water + 0.1% formic acid, B: acetonitrile + 0.1% formic acid; flow rate 0.8 ml/min) on an Agilent RRHD EclipsePlus 95 Å C18 column (2.1 × 50 mm, 1.8 µm, 1200 bar). EIC values were obtained as above.

### FPLC purification of PE-vpl2

Apoplast extracts from 54 leaves of *N. benthamiana* heterologously producing PE-*vpl2* were prepared as above and dialyzed into 20 mM sodium succinate, pH 5.5, using 20 K MWCO 15-ml Slide Cassette G2 diafiltration cassettes. Protein was purified using a 1 ml HiTrap Q HP column (GE Healthcare Systems) running a gradient from 0 to 0.25 M sodium chloride at 1 ml min^−1^ (Supplementary Fig. 10c). Fractions with veratryl alcohol were pooled and concentrated for use in experiments.

### Methylated DHP lignin preparation

Unmethylated DHP lignin was prepared as described previously^29^. 100 mg sinapyl alcohol and 17 mg coniferyl alcohol were dissolved in 2 ml acetone and diluted into 20 ml 10 mM sodium phosphate (pH 6.5) containing 60 purpurogallin units of peroxidase from horseradish (Serva) (solution 1). Separately, 55 μl 30% hydrogen peroxide was diluted into 20 ml 10 mM sodium phosphate pH 6.5 (solution 2). Solutions 1 and 2 were slowly added using a syringe pump to a round-bottom flask containing 1.5 mg vanillyl alcohol in 10 ml 10 mM sodium phosphate pH 6.5. Addition of the solutions was performed at 2 ml/h at room temperature while stirring under argon, shielded from light using aluminum foil. The reaction was allowed to proceed for a total of 24 h. The reaction products were centrifuged (3200 rpm, 30 min, 4 °C) and washed twice with 50 ml water before lyophilization.

Methylation was performed using trimethylsilyldiazomethane (TMSD) as previously described^54^. Twenty milligrams of DHP lignin was dissolved in 360 μl *N,N*-dimethylformamide in a 1.6 ml SafeLock Eppendorf microcentrifuge tube. Forty microliters of methanol, 24.26 μl *N,N*-diisopropylethylamine, and 64 μl TMSD (2.2 M in *n*-hexanes) were sequentially added. The reaction was performed at room temperature with rotation for 18 h. The reaction contents were transferred across 6 microcentrifuge tubes and each diluted 17.5-fold with water before centrifugation (21,000 × *g*, 10 min). The pellets were washed four times with 1 ml water before lyophilization.

### DHP lignin depolymerization

Depolymerization reactions contained 0.66 μM FPLC-purified PE-vpl2, 200 μg/ml DHP lignin, 10 mM veratryl alcohol, and 0.25% *v/v* Tween-20 in 10 mM sodium acetate, pH 4.5. Enzyme was replaced with an equal volume of buffer (15 mM sodium succinate and 10% *v/v* glycerol, pH 5.6) for reactions without enzyme. One hundred micromolar hydrogen peroxide was added every 1.5 h for 6 additions in total. Reactions were performed with rotation in Protein Lo-Bind SafeLock microcentrifuge tubes (Eppendorf) at room temperature. Conditions were the same for reactions involving unmethylated and methylated DHP lignin.

### Gel permeation chromatography

Depolymerization reactions were lyophilized and reconstituted in 125 μl *N,N*-dimethylformamide containing 0.1 M lithium bromide. Samples were clarified (21,000 × *g*, 10 min) and 60 μl injected on two PSS-PolarSil columns in series with *N,N*-dimethylformamide containing 0.1 M lithium bromide as the running solvent and a flow rate of 1 ml/min. Lignin elution was monitored using a diode-array detector at 310 nm.

### Methoxybenzene activity testing

Reactions contained 0.4 mM methoxybenzene substrate, 0.66 μΜ FPLC-purified PE-*vpl2* or commercial peroxidase from horseradish (Serva), and 0.2 mM hydrogen peroxide in 50 mM sodium tartrate, pH 3.5. Enzyme concentrations were normalized using spectrophotometric measurements of heme absorbance at 410 nm and extinction coefficients of 133 and 105 mM^−1^ cm^−1^ for PE-vpl2 and horseradish peroxidase, respectively. Reactions were carried out at room temperature for 1 h before injection of 5 μl on an Agilent 6520 Q-TOF LC-MS running in positive mode with an 25-min water-acetonitrile gradient (0 min, 97% A; 1 min, 97% A; 20 min, 3% A; 22 min, 3% A; 22.5 min, 97% A; 25 min, 97% A; A: water + 0.1% formic acid, B: acetonitrile + 0.1% formic acid; flow rate 0.4 ml/min) on an Agilent RRHD EclipsePlus 95 Å C18 column (2.1 × 50 mm, 1.8 µm, 1200 bar). For all substrates except 1,4-dimethoxybenzene, substrate conversion was measured as difference in MS peak area corresponding to the methoxybenzene substrate between enzyme and no-enzyme conditions. For 1,4-dimethoxybenzene, substrate conversion was measured using UV peak area at 270 nm. In experiments involving 1,2,3,5-tetramethoxybenzene, substrate conversion extent was found to be dependent on the concentration of horseradish peroxidase used. Given this sensitivity to enzyme concentration, our data should not be used to estimate the absolute redox potential of either enzyme assayed here. Redox potentials of methoxybenzene substrates used here were previously determined in non-aqueous conditions as polarographic half-wave potentials using a rotating platinum electrode^50^.

### Statistics and reproducibility

All in vitro enzyme reactions were separately assembled and performed in triplicate. For protein production in *N. benthamiana*, three leaves on three separately grown plants were chosen at random as biological replicates. Plants were selected from the same batch planted on the same date. Spectroscopic activity measurements were performed as technical triplicates for each individual leaf in *N. benthamiana* and activity levels were calculated as the average of the three individual leaves. GFP-expressing leaf controls were included in each round of protein production in *N. benthamiana* to control for variability between plant batches. For protein production in *S. cerevisiae*, three individual colonies were selected at random from each vector transformation as biological replicates and were used as such for spectroscopic activity measurements. All data points represent the arithmetic mean of three independent replicates and error bars represent one standard deviation. A two-tailed *t*-test with a *p*-value of 0.05 was used to determine statistical significance relative to GFP controls in activity level measurements after exclusion of outliers.

### Reporting summary

Further information on research design is available in the Nature Research Reporting Summary linked to this article.

## Supplementary information

SUPPLEMENTARY INFORMATION

Description of Supplementary Files

Supplementary Data 1

Reporting Summary

## Data Availability

The source data for graphs and charts is available as Supplementary Data 1. Any remaining information can be obtained from the corresponding author upon reasonable request.
