# Supplementary material for: A plant host, Nicotiana benthamiana, enables the production and study of fungal lignin-degrading enzymes
Source: Commun Biol. 2021 Sep 1;4:1027. doi: 10.1038/s42003-021-02464-9 (PMC8410833; doi:10.1038/s42003-021-02464-9)
Supplement: Supplementary file 1 — SUPPLEMENTARY INFORMATION [file 42003_2021_2464_MOESM1_ESM.pdf]

Supporting Information

Table of Contents

Supplementary Figure 1. BJ5465 versus JHY693 yeast secretion performance
Supplementary Figure 2. Testing of promoters and ER signal peptides in *S. cerevisiae*
Supplementary Figure 3. Laccase and pyranose oxidase production in *S. cerevisiae* and *N. benthamiana*
Supplementary Figure 4. ABTS activity of white-rot peroxidases tested in *S. cerevisiae*
Supplementary Figure 5. Yeast supernatant inhibition of commercial LiP activity on veratryl alcohol
Supplementary Figure 6. Schematic of enzyme extraction from *N. benthamiana*
Supplementary Figure 7. Summary of raw peroxidase activity of culture supernatant of *S. cerevisiae* and apoplast extracts of *N. benthamiana* towards ABTS and veratryl alcohol or Mn(II), without subtracting background activity of corresponding GFP-expressing controls.
Supplementary Figure 8. Testing of different ER signal peptides for PE-vpl2 production in *N. benthamiana*.
Supplementary Figure 9. *In vitro* oxidation of unmethylated lignin by heterologous PE-vpl2 and comparison of oxidation of non-phenolic and phenolic versions of a model lignin dimer.
Supplementary Figure 10. Total protein gel, Western blotting, and FPLC purification of enzymes produced in *N. benthamiana*.
Supplementary Figure 11. Negative controls for coupling experiments
Supplementary Figure 12. Product formation and dimer cleavage extent by direct and Mn(III)-mediated oxidation
Supplementary Figure 13. Glucose oxidase stability as a function of pH and improvement in substrate conversion by addition of catalase
Supplementary Figure 14. Western blotting of enzymes secreted by *S. cerevisiae*
Supplementary Figure 15. PE-aao(FX9) and PE-vpl2 activities on benzyl alcohol as a substrate
Supplementary Figure 16. Inhibitory effects of MES buffer on veratryl alcohol oxidation by commercial LiP

Supplementary Table 1. Heme concentration of diafiltrated extracts
Supplementary Table 2. List of strains used
Supplementary Table 3. List of vectors used
Supplementary Table 4. List of genes used
Supplementary Table 5. List of ER signal peptides used
Supplementary Table 6. List of antibody epitope tags used

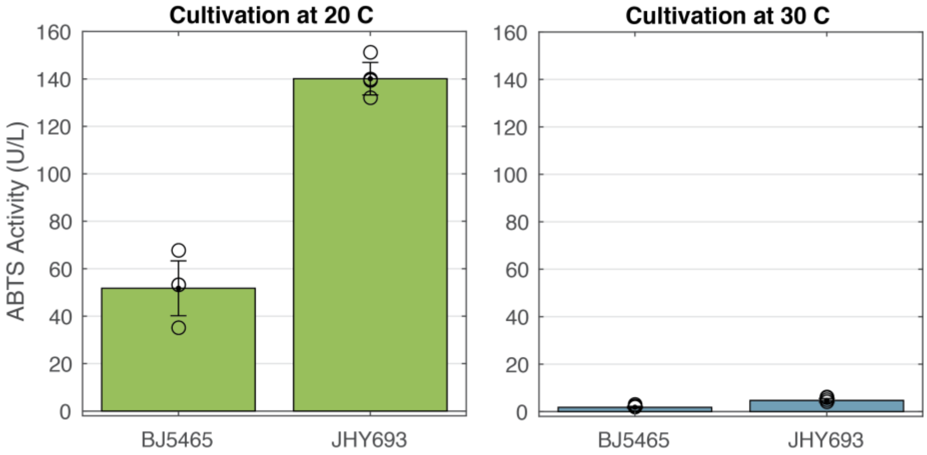

Supplementary Figure 1. BJ5465 versus JHY693 yeast secretion performance. The production of horseradish peroxidase (HRP) was compared in *S. cerevisiae* strains BJ5465[1] and JHY693[2] at 20 and 30 degrees Celsius. ABTS activity was determined as described in Methods.

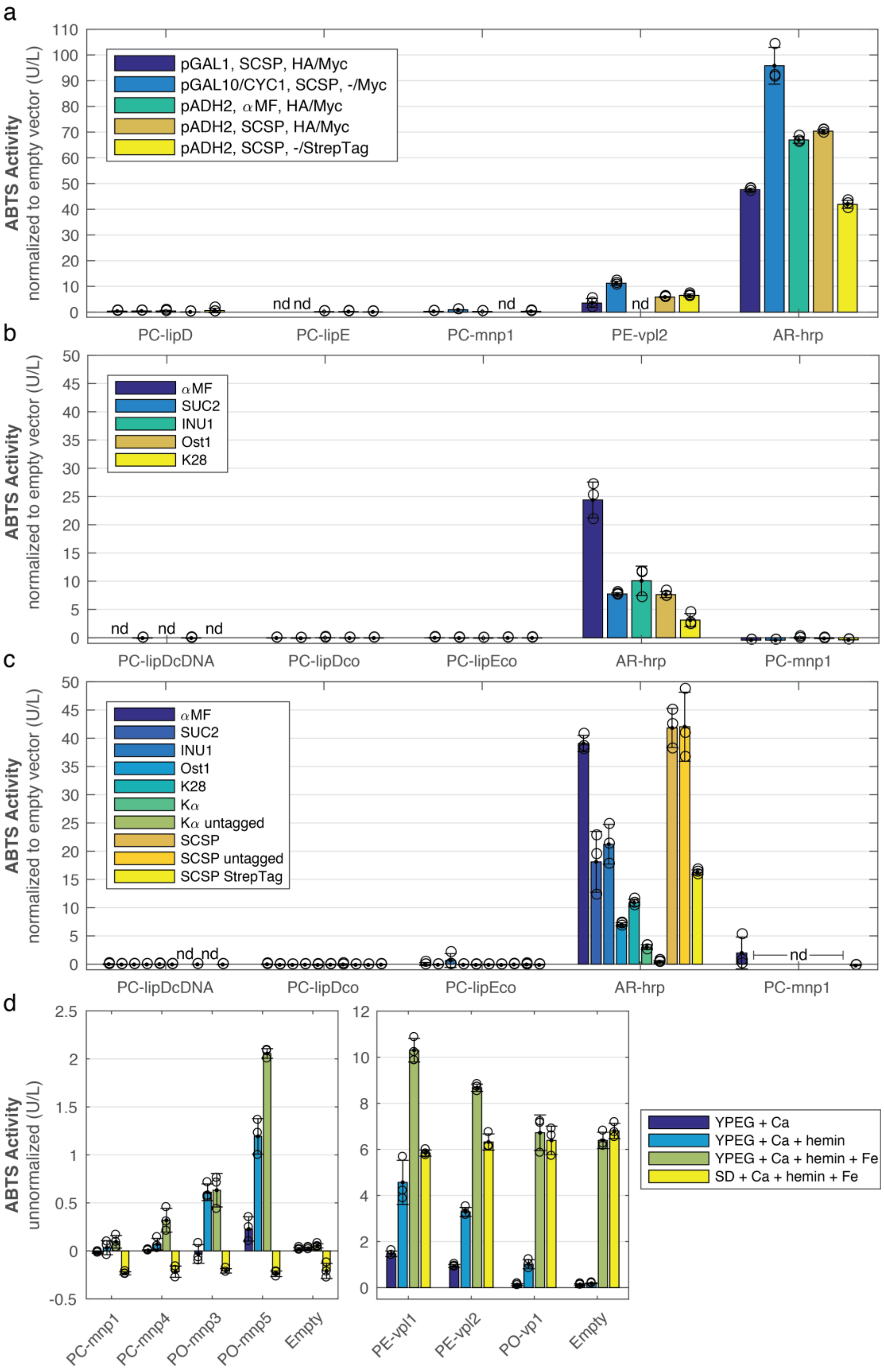
a
ABTS Activity
normalized to empty vector (U/L)
pGAL1, SCSP, HA/Myc
pGAL10/CYC1, SCSP, -/Myc
pADH2, αMF, HA/Myc
pADH2, SCSP, HA/Myc
pADH2, SCSP, -/StrepTag
nd
PC-lipD
PC-lipE
PC-mnp1
PE-vpl2
AR-hrp
b
αMF
SUC2
INU1
Ost1
K28
PC-lipDcDNA
PC-lipDco
PC-lipEco
AR-hrp
PC-mnp1
c
αMF
SUC2
INU1
Ost1
K28
Kα
Kα untagged
SCSP
SCSP untagged
SCSP StrepTag
d
ABTS Activity
unnormalized (U/L)
PC-mnp1
PC-mnp4
PO-mnp3
PO-mnp5
Empty
PE-vpl1
PE-vpl2
PO-vp1
Empty
YPEG + Ca
YPEG + Ca + hemin
YPEG + Ca + hemin + Fe
SD + Ca + hemin + Fe

Supplementary Figure 2. Testing of promoters and ER signal peptides in *S. cerevisiae.* a) Comparison of promoter, signal peptides, and antibody affinity tags on production of lignin-degrading peroxidases from *S. cerevisiae* from high-copy 2µ expression cassettes. **b)** Comparison of signal peptide choice in the production of peroxidases in *S. cerevisiae* from the low-copy CEN/ARS expression cassette[3] pRS415-ADH2. **c)** Comparison of signal peptide choice in the production of peroxidases in *S. cerevisiae* from the high-copy 2µ expression cassette pCHINT2AL[2]. **d)** Optimization of media conditions for peroxidase production from pL231 expression cassette in *S. cerevisiae*. YPEG, rich media; SD, synthetic defined media. Signal peptides: αMF, alpha-mating factor, evolved variant appS4[4]; SUC2, *S. cerevisiae* invertase; INU1, *K. marxianus* inulinase; Ost1, *S. cerevisiae* pre-*Ost1*-pro-αMF fusion[5]; K28, K28 killer toxin; Kα, killer toxin alpha subunit; SCSP, synthetic consensus signal peptide[6]. PC, *P. chrysosporium*; PE, *P. eryngii*; PO, *P. ostreatus*; AR, *A. rusticana*; cDNA, sequence from cDNA; co, sequence codon-optimized for expression in *S. cerevisiae*. Antibody affinity tags: HA, hemagglutinin; Myc: c-myc. nd: not determined. ABTS activity was determined as described in Methods. Error bars represent one standard deviation in activity levels of three biological replicates.

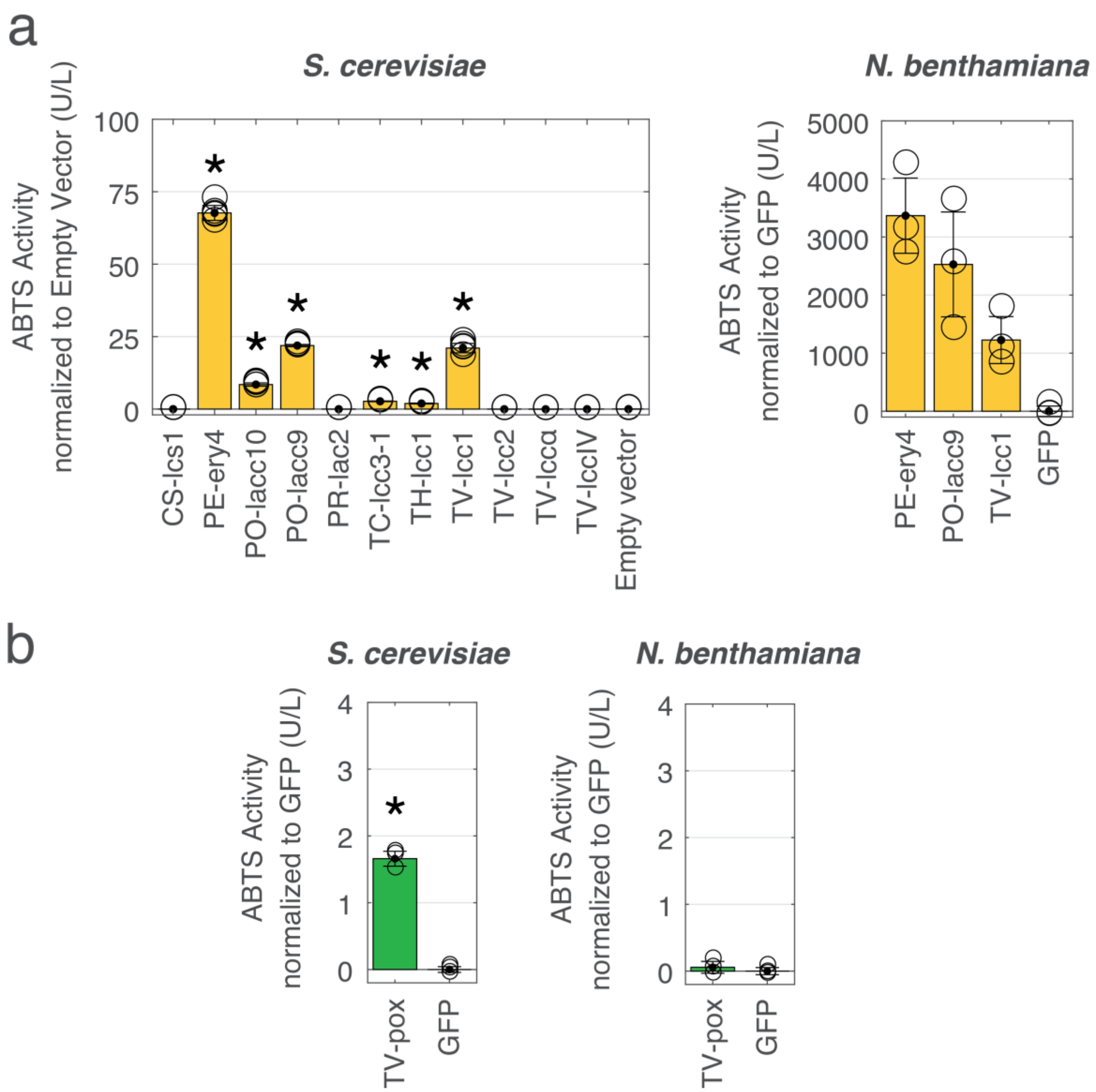

Supplementary Figure 3. Laccase and pyranose oxidase production in *S. cerevisiae* and *N. benthamiana*. a) ABTS activity of supernatant of *S. cerevisiae* and of crude apoplast extracts of *N. benthamiana* plants producing laccases. Error bars for S. cerevisiae measurements represent one standard deviation in activity determined from five biological replicates; asterisks indicate statistical significance relative to a no-vector control ($p < 0.05$). Error bars for N. benthamiana represent one standard deviation in activity determined from three individual leaves as biological replicates. **b)** ABTS activity of supernatant of *S. cerevisiae* and of crude apoplast extracts of *N. benthamiana* plants producing a pyranose oxidase from *T. versicolor* (TV-pox). Error bars for represent one standard deviation in activity determined from three biological replicates; asterisk indicates statistical significance relative to a GFP-expressing control ($p < 0.05$).

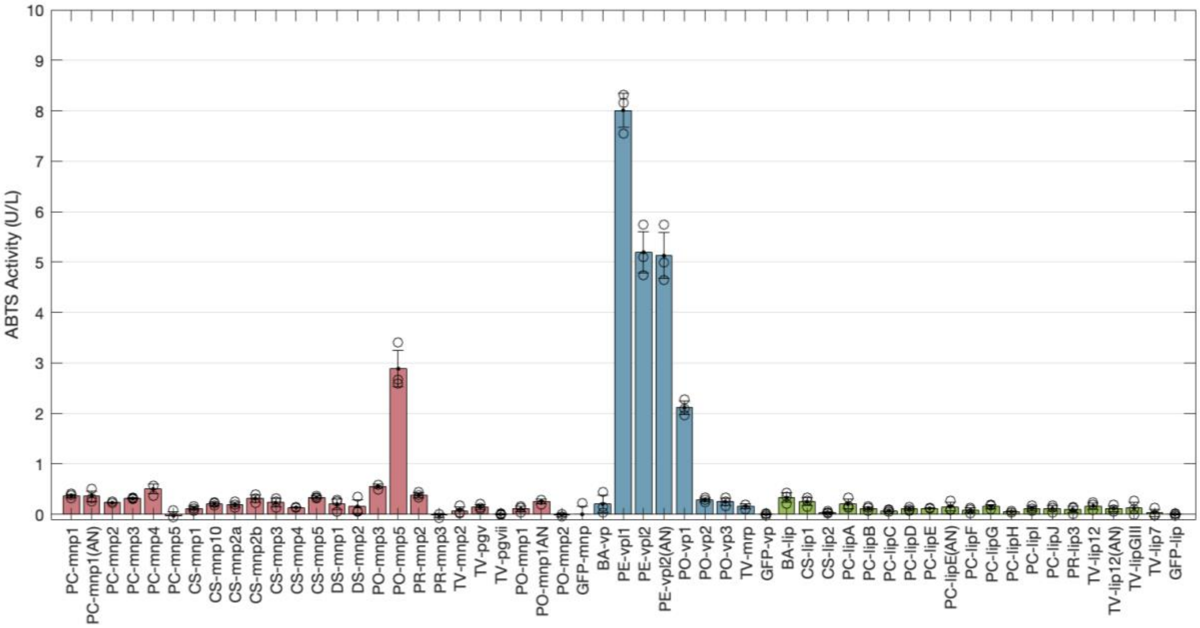

Supplementary Figure 4. ABTS activity of all white-rot peroxidases tested in *S. cerevisiae*. Activity was determined as described in Methods. PC, *P. chrysosporium*; CS, *C. subvermispora*; DS, *D. squalens*; PE, *P. eryngii*; PO, *P. ostreatus*; PR, *P. radiata*; TV, *T. versicolor*; BA, *B. adusta*. GFP corresponds to GFP-expressing strains tested under the corresponding conditions appropriate for the peroxidase type. (AN) refers to gene codon optimization for *Aspergillus nidulans*. Error bars represent one standard deviation in activity levels of three biological replicates.

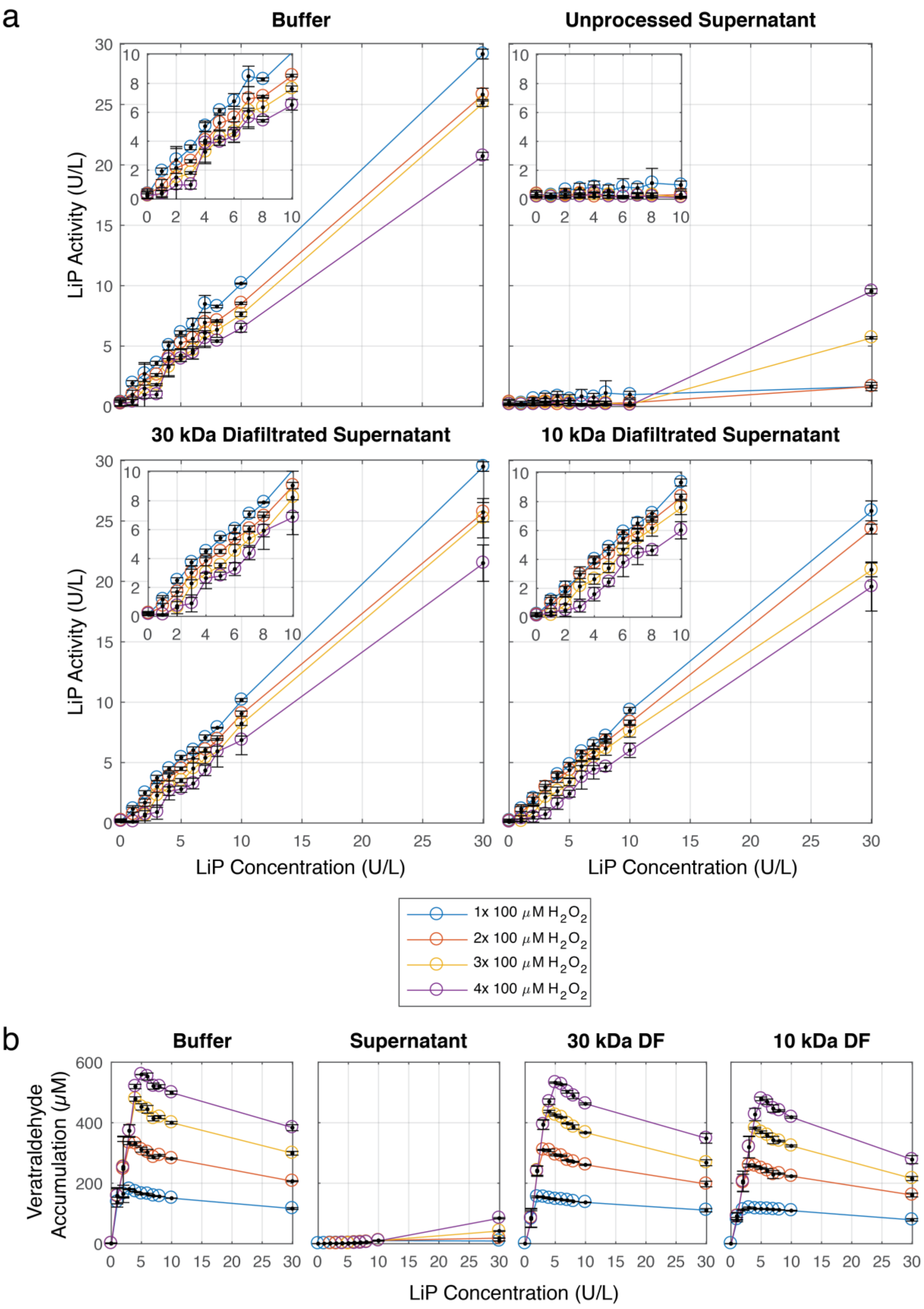

a
Buffer
Unprocessed Supernatant
30 kDa Diafiltrated Supernatant
10 kDa Diafiltrated Supernatant
LiP Activity (U/L)
LiP Concentration (U/L)
1x 100 μM H2O2
2x 100 μM H2O2
3x 100 μM H2O2
4x 100 μM H2O2
b
Buffer
Supernatant
30 kDa DF
10 kDa DF
Veratraldehyde Accumulation (μM)
LiP Concentration (U/L)

Supplementary Figure 5. Yeast supernatant inhibition of commercial LiP activity on veratryl alcohol. Commercial lignin peroxidase (Sigma) was assayed for activity on veratryl alcohol in the context of 10% *v/v* buffer, unprocessed supernatant from *S. cerevisiae* expressing GFP, supernatant diafiltrated 10000-fold through 30 kDa and 10 kDa size-exclusion centrifugal filters with 20 mM sodium acetate, pH 6.0. LiP activity on veratryl alcohol was measured by absorbance at 310 nm indicating formation of veratraldehyde as a product (ε = 9300 1/M 1/cm)[7]. Assays were initiated using 100 µM hydrogen peroxide, which was successively added three more times after full peroxide consumption as indicated by constant absorbance readings. **a)** Observed LiP activity as a function of LiP concentration in the reaction. **b)** Accumulation of veratraldehyde as a function of LiP concentration. Unprocessed yeast supernatant inhibited the formation of veratraldehyde at low LiP concentrations, which was only observed after the third addition of peroxide, presumably after full conversion of inhibiting compounds. Diafiltration eliminated the observed inhibition. Error bars represent one standard deviation of triplicate activity assays.

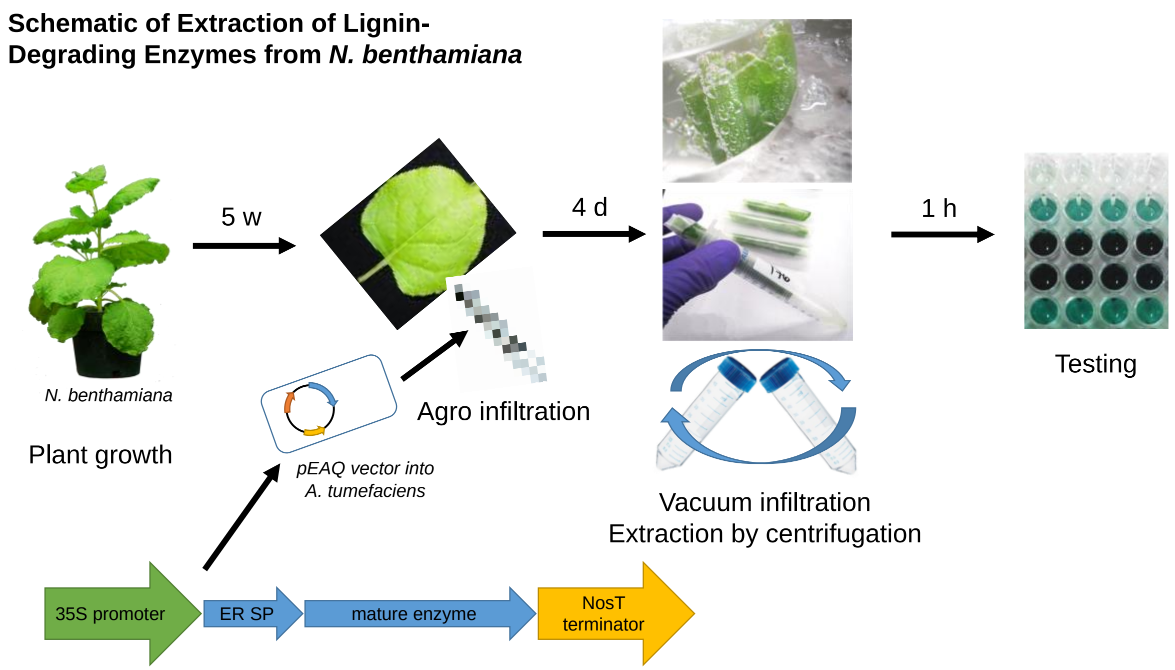

Supplementary Figure 6. Schematic of enzyme extraction from *N. benthamiana*. Crude apoplast extracts are produced from 5-week-old *N. benthamiana* plants transiently transformed with *Agrobacterium tumefaciens* harboring pEAQ expression vectors. Protocol adapted from O'Leary *et al*[8].

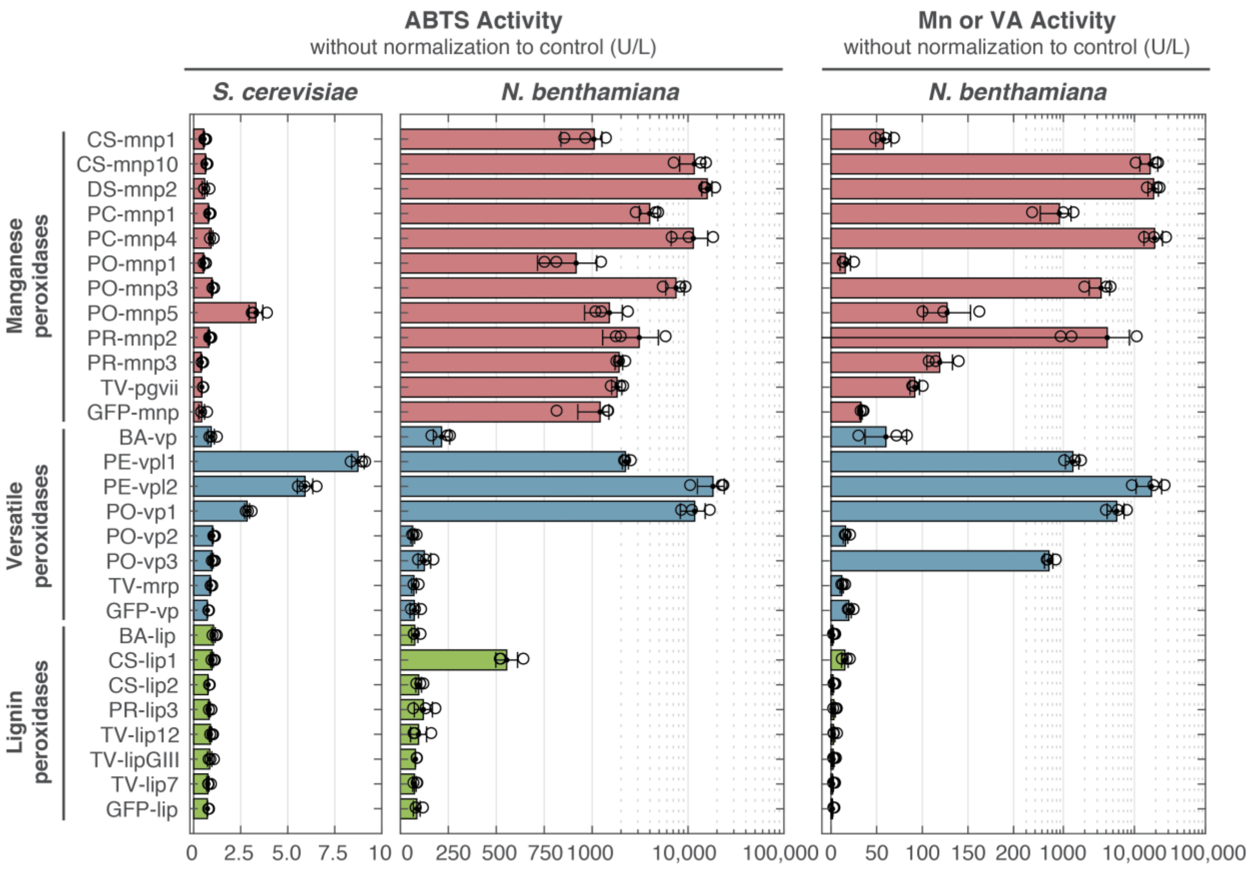

Supplementary Figure 7. Summary of raw peroxidase activity of culture supernatant of *S. cerevisiae* and apoplast extracts of *N. benthamiana* towards ABTS and veratryl alcohol or Mn(II), without subtracting background activity of corresponding GFP-expressing controls. Lignin peroxidases are shown in green, versatile peroxidases in blue, and manganese peroxidases in red. Manganese peroxidases were assayed for ABTS activity using 4 mM ABTS, 100 µM H_2O_2, 1 mM $MnSO_4$, 50 mM sodium malonate, pH 4.5; and for Mn/veratryl alcohol activity using 1 mM $MnSO_4$, 100 µM H_2O_2, 50 mM sodium malonate, pH 4.5. Versatile peroxidases were assayed for ABTS activity using 4 mM ABTS, 100 µM H_2O_2, 50 mM sodium tartrate, pH 3.5; and for Mn/veratryl alcohol activity using 1 mM $MnSO_4$, 100 µM H_2O_2, 50 mM sodium malonate, pH 4.5. Lignin peroxidases were assayed for ABTS activity using 4 mM ABTS, 100 µM H_2O_2, 50 mM sodium tartrate, pH 3.5; and for Mn/veratryl alcohol activity using 20 mM veratryl alcohol, 100 µM H_2O_2, 50 mM sodium tartrate, pH 3.5. The high background ABTS activity of GFP control in the manganese peroxidases relative to that of the other two GFP controls was reproducible across several experimental batches and is likely a result of the higher pH used to assay manganese peroxidase extracts for ABTS activity. Activities represent 1 µM oxidized product formed min^{-1} l^{-1}. Activities were measured of three different leaves in *N. benthamiana* 5 days after *Agrobacterium* infiltration, or three biological replicates for *S. cerevisiae* after 2 days of cultivation. Data points represent the average of three independent reaction replicates with error bars calculated as one standard deviation. GFP-expressing control samples for each set of enzymes are designated GFP-mnp, GFP-vp, and GFP-lip. BA = *Bjerkandera adusta*, CS = *Ceriporiopsis (Gelatoporia)*

subvermispora, PC = *Phanerochaete chrysosporium*, PE = *Pleurotus eryngii*, PO = *Pleurotus ostreatus*, PR = *Phlebia radiata*, TC = *Trametes (Pycnoporus) cinnabarinus*, TV = *Trametes versicolor*.

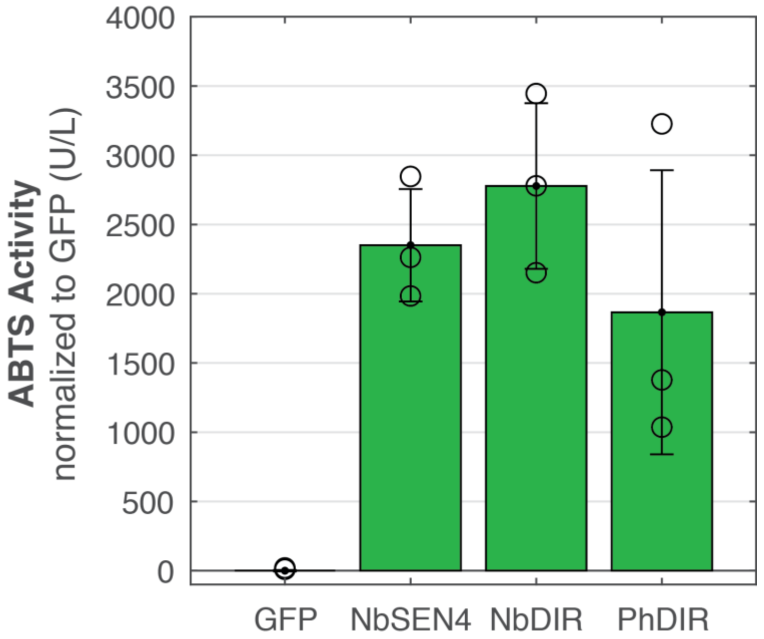

Supplementary Figure 8. Testing of different ER signal peptides for PE-vpl2 production in *N. benthamiana*. pEAQ expression cassettes harboring the mature PE-vpl2 sequence were fused with signal peptides derived from xyloglucan endotransglucoslase/hydrolase (*NbSEN4*, UNIPROT A0A1Q1N6K4) of *N. benthamiana* or dirigent protein (*NbDIR*, UNIPROT Q0WYB7) of N. benthamiana, and compared to that of dirigent protein (*PhDIR*) of *P. hexandrum*, which was used for all other expression cassettes in *N. benthamiana*. ABTS activity was measured as described in Methods.

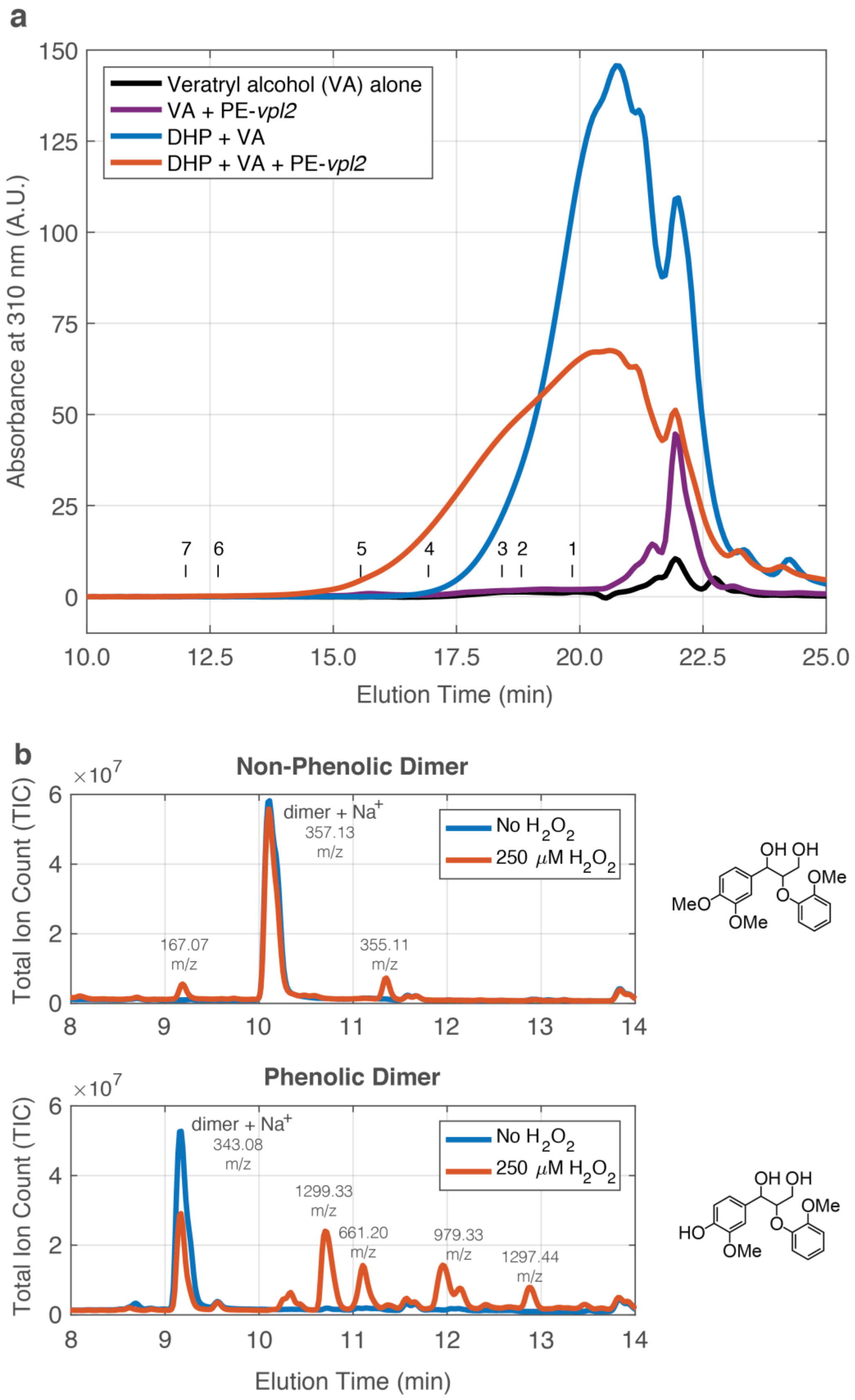
a
Veratryl alcohol (VA) alone
VA + PE-vpl2
DHP + VA
DHP + VA + PE-vpl2
Absorbance at 310 nm (A.U.)
Elution Time (min)
7
6
5
4
3
2
1
b
Non-Phenolic Dimer
Phenolic Dimer
Total Ion Count (TIC)
×10^7
dimer + Na+
357.13 m/z
167.07 m/z
355.11 m/z
343.08 m/z
1299.33 m/z
661.20 m/z
979.33 m/z
1297.44 m/z
No H2O2
250 μM H2O2
OH OH
OMe
MeO
HO
O
Elution Time (min)

Supplementary Figure 9. *In vitro* oxidation of unmethylated lignin by heterologous PE-vpl2 and comparison of oxidation of non-phenolic and phenolic versions of a model lignin dimer. **a)** Gel permeation chromatography (GPC) of unmethylated DHP lignin subjected to oxidation by heterologous FPLC-purified PE-vpl2 extracted from *N. benthamiana*. *In vitro* conditions involved 200 μg/ml unmethylated DHP lignin, 10 mM veratryl alcohol, 0.25% Tween-20, and 0.66 μM purified enzyme in 10 mM sodium acetate, pH 4.5. 100 μM hydrogen peroxide was added every 1.5 hours for a total of 600 μM. Control reactions had enzyme omitted (DHP alone), lignin omitted (veratryl alcohol + PE-vpl2), and both enzyme and lignin omitted (veratryl alcohol alone). Absorbance traces shown here are representative of reaction duplicates. The molecular weights of polystyrene standards used for calibration were as follows: *1*, 1.37 kg/mol; *2*, 2.93 kg/mol; *3*, 4.43 kg/mol; *4*, 10.1 kg/mol; *5*, 21.7 kg/mol; *6*, 139 kg/mol; *7*, 281 kg/mol. **b)** Liquid chromatography mass spectrometry (LC-MS) analysis of PE-vpl2 oxidation of a model non-phenolic and phenolic β-O-4 lignin dimer. Oxidation of phenolic dimer gave rise to new signatures with higher mass values corresponding to oligomers, whereas oxidation of non-phenolic dimer gave rise to signatures with lower mass values, corresponding to veratraldehyde and dehydrodimer degradation products. Reactions contained 500 μM dimer, 250 μM hydrogen peroxide, and 0.53 μM FPLC-purified PE-vpl2 in 25 mM sodium acetate, pH 4.5. Reactions were incubated for 1 hour at room temperature before injection of 1 μl on an Agilent 6545 Q-TOF LC-MS instrument.

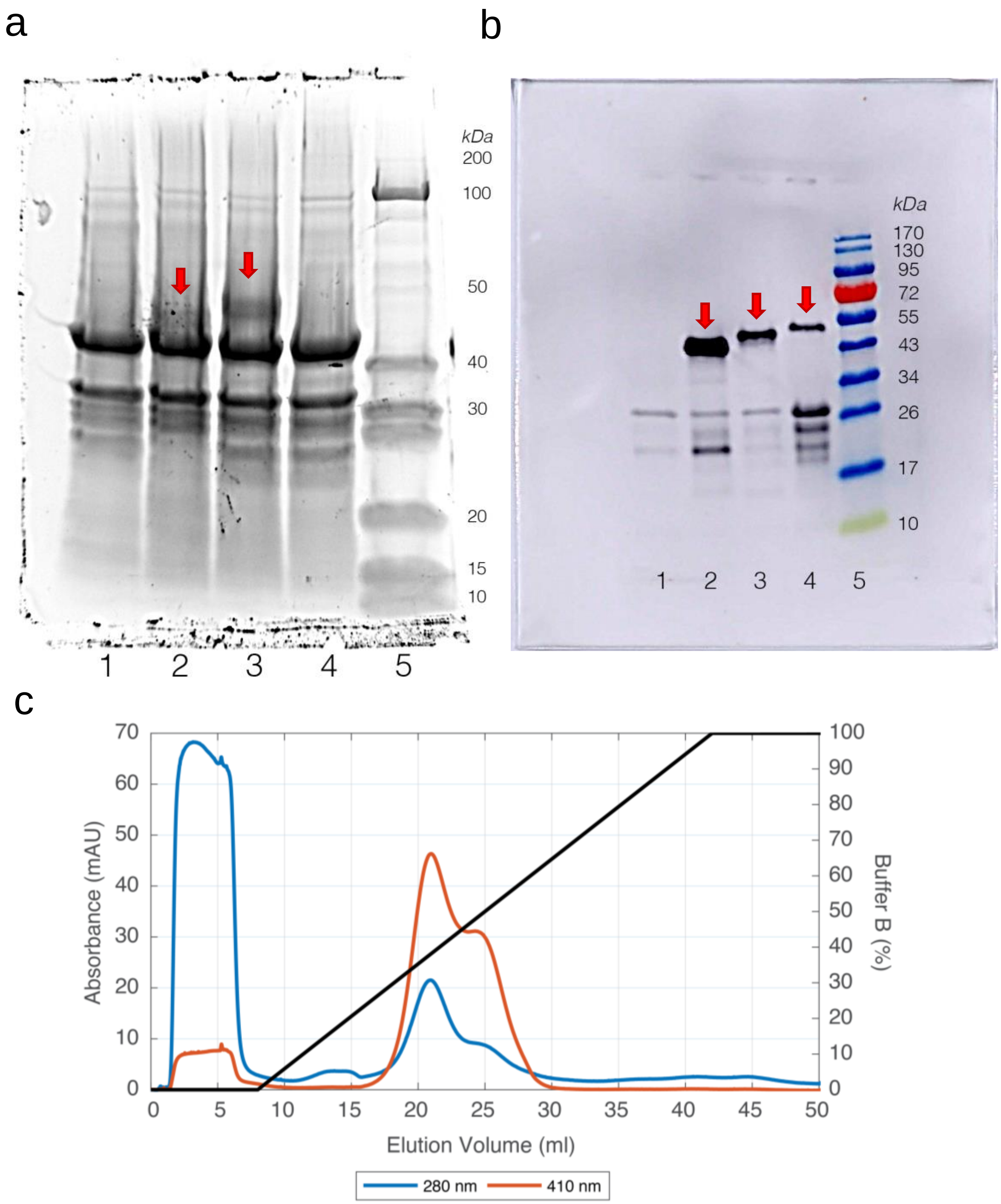

Supplementary Figure 10. Total protein gel, Western blotting, and FPLC purification of enzymes produced in *N. benthamiana*. a) 5.55 µg of total protein (as measured by Bradford assay[9]) of diafiltrated apoplast extracts of *N. benthamiana* were analyzed by Flamingo (BioRad) staining. Lane 1, GFP control; lane 2, PE-vpl2; lane 3, PC-mnp1; lane 4, CS-lip1; lane 5, protein ladder (Fisher). Red arrows indicate expected bands corresponding to PE-vpl2 and PC-mnp1, respectively; CS-lip1 is presumably too faint to be detected. **b)** 5 µl of diafiltrated apoplast extracts of *N. benthamiana* were analyzed by Western blotting of C-terminal Myc tag of lignin-degrading

peroxidases. Lane 1, GFP control (547 μg/ml total protein); lane 2, PE-vpl2 (306 μg/ml); lane 3, PC-mnp1 (370 μg/ml); lane 4, CS-lip1 (1781 μg/ml); lane 5, protein ladder (Fisher). Red arrows indicate expected bands corresponding to PE-vpl2, PC-mnp1, and CS-lip1. The expected molecular weight of the mature enzymes including affinity tags is between 37, 40 and 38 kDa, respectively. **c)** FPLC purification chromatographs of PE-vpl2 heterologously produced in *N. benthamiana*. Absorbance at 280 nm and 410 nm were monitored during elution by a gradient from 0 mM to 250 mM sodium chloride. Absorbance at 410 nm is indicative of eluted protein containing a heme cofactor. Extraneous proteins contained in the apoplast extract were not bound by the affinity column and eluted before the gradient. No activity towards veratryl alcohol was observed for this elution peak. A broad elution peak between 20 and 30 ml was observed with high absorbance at 410 nm relative to 280 nm and exhibited strong activity towards veratryl alcohol, indicating that the peak corresponded to purified PE-vpl2.

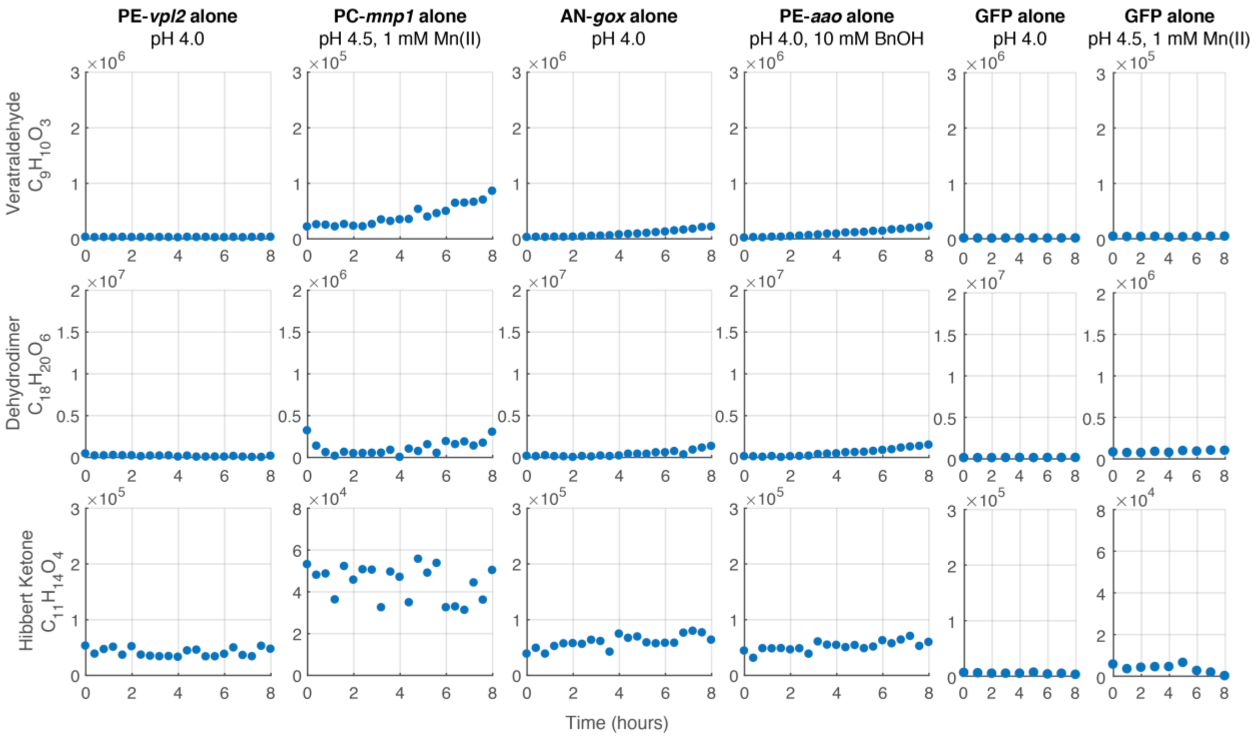

Supplementary Figure 11. Negative controls for coupling experiments. Diafiltrated apoplast extracts of PE-vpl2 and PC-mnp1 from *N. benthamiana* were individually assayed for activity towards a model β-O-4 lignin dimer under conditions corresponding to those used in Figure 3 except without the addition of peroxide-generating enzymes. Commercially-available glucose oxidase (AN-gox) and diafiltrated apoplast extract of PE-aao(FX9) from *N. benthamiana* were tested in the same way without the addition of lignin-degrading peroxidases. Apoplast extract of GFP-expressing *N. benthamiana* was tested in the same way with and without Mn(II), except with lower reaction sampling frequency and 10 mM dimer instead of 20 mM.

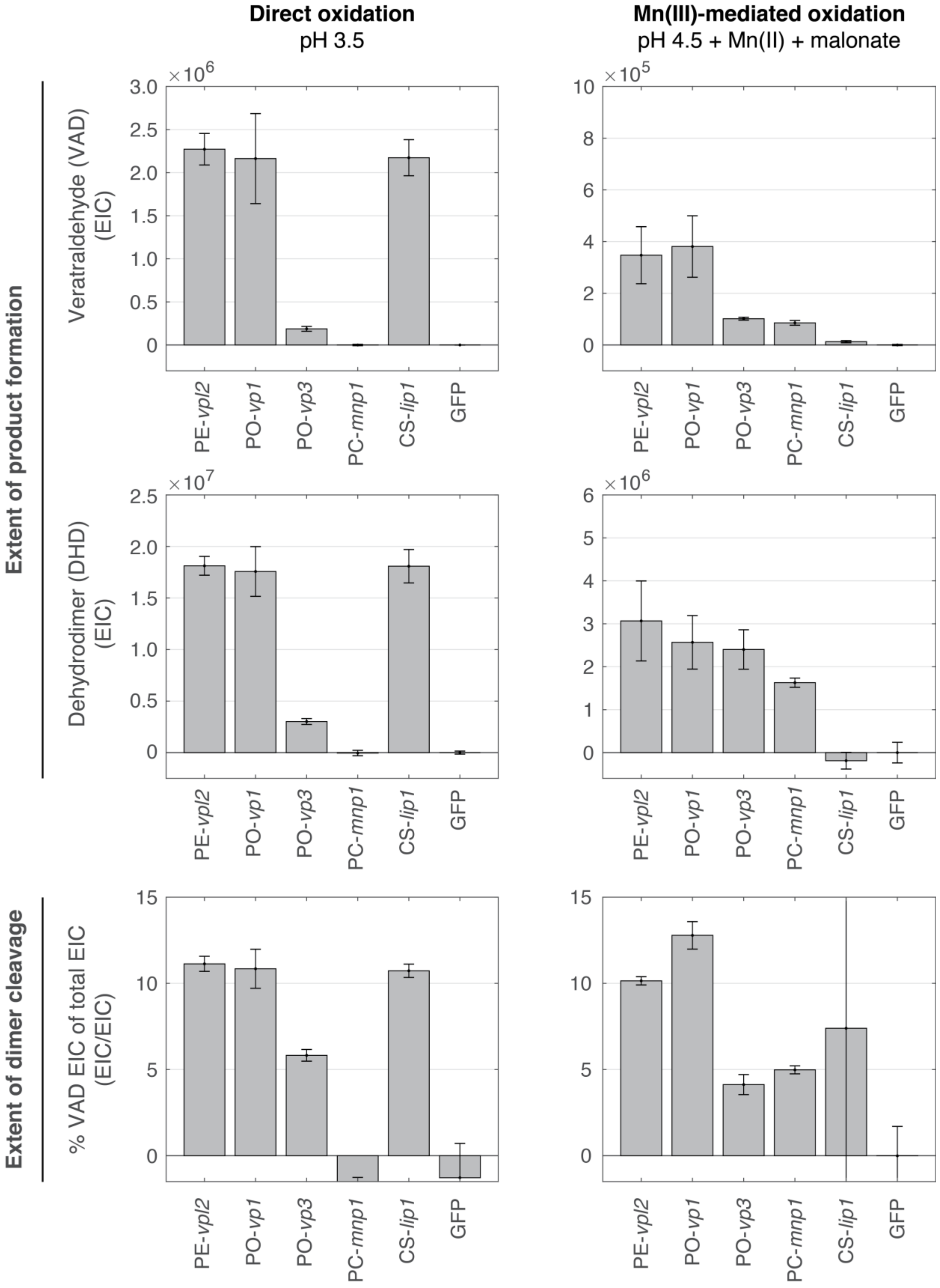

Direct oxidation
pH 3.5
Mn(III)-mediated oxidation
pH 4.5 + Mn(II) + malonate
Extent of product formation
Extent of dimer cleavage
Veratraldehyde (VAD) (EIC)
Dehydrodimer (DHD) (EIC)
% VAD EIC of total EIC (EIC/EIC)
×10^6
×10^5
×10^7
×10^6
PE-vpl2
PO-vp1
PO-vp3
PC-mnp1
CS-lip1
GFP

Supplementary Figure 12. Product formation and dimer cleavage extent by direct and Mn(III)-mediated oxidation. Reactions were performed as described in Methods. Diafiltrated apoplast extracts from heterologous *N. benthamiana* containing peroxidases were coupled with glucose oxidase from *A. niger* in dimer oxidation reactions containing either 50 mM sodium tartrate, pH 3.5, or 50 mM sodium malonate, pH 4.5, and 1 mM $MnSO_4$, representing conditions favoring direct and Mn(III)-mediated oxidation, respectively. Dimer cleavage extent (bottom row) was determined as the proportion of EIC corresponding to veratraldehyde relative to total EIC corresponding to the sum of veratraldehyde and dehydrodimer and is represented as a percentage. The EIC data used for this calculation is net of the EIC detected for the GFP samples, and cleavage extent was calculated for each replicate individually before averaging. Data bars represent the average of three independent replicate reactions, and error bars represent one standard deviation.

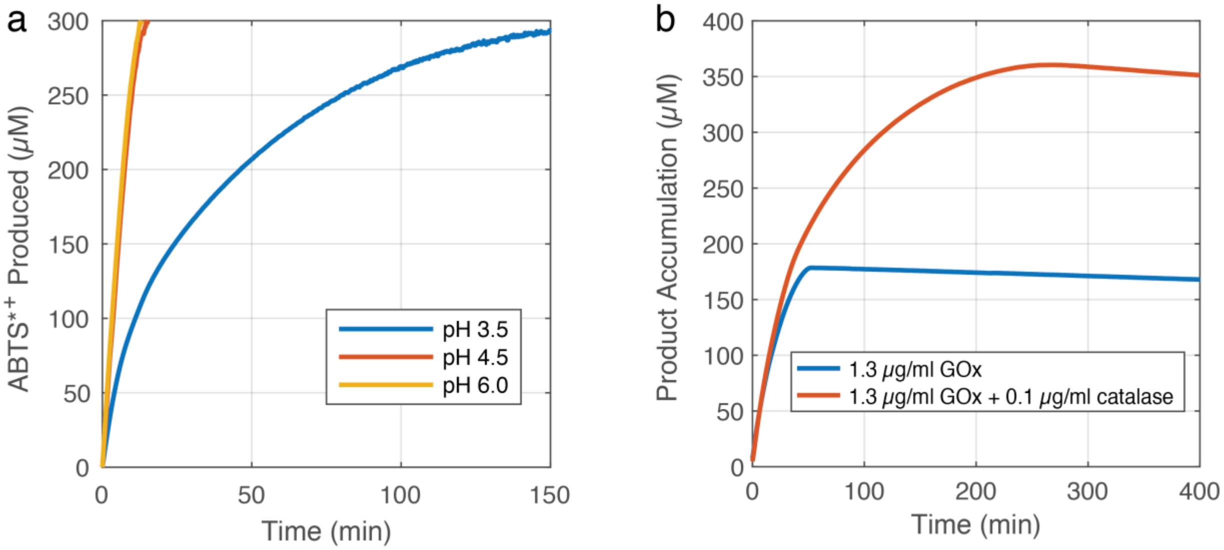

Supplementary Figure 13. Glucose oxidase stability as a function of pH and effects of catalase on product accumulation in coupled dimer oxidation. a) Glucose oxidase stability was tested as a function of pH with ABTS oxidation as a readout catalyzed by horseradish peroxidase (HRP). Reactions involved 0.574 ng/µl commercial glucose oxidase (Sigma), 4 mM ABTS, 25 ng/µl commercial HRP (Serva), 50 mM sodium tartrate, pH 3.5, or sodium malonate, pH 4.5, or sodium acetate, pH 6.0, and 0.4% *w/v* D-glucose. Reactions were performed at 25 C and ABTS oxidation was measured spectroscopically at 414 nm using an extinction coefficient of 36000 1/M 1/cm. The reactions at pH 4.5 and 6.0 saturated the photodetector while the reaction at pH 3.5 did not, highlighting the instability of glucose oxidase under acidic conditions. **b)** Model dimer conversion by a coupled system of heterologous PE-vpl2 extracted from *N. benthamiana* and commercial glucose oxidase (Sigma) was monitored at 310 nm in the presence and absence of catalase (0.1 ng/µl, Sigma). Reactions involved 20 mM dimer in 50 mM sodium tartrate, pH 3.5, containing 0.4% *w/v* D-glucose with 1.3 ng/µl commercial glucose oxidase. Product accumulation was measured using the extinction coefficient of veratraldehyde at 310 nm (9300 M^{-1} cm^{-1}).

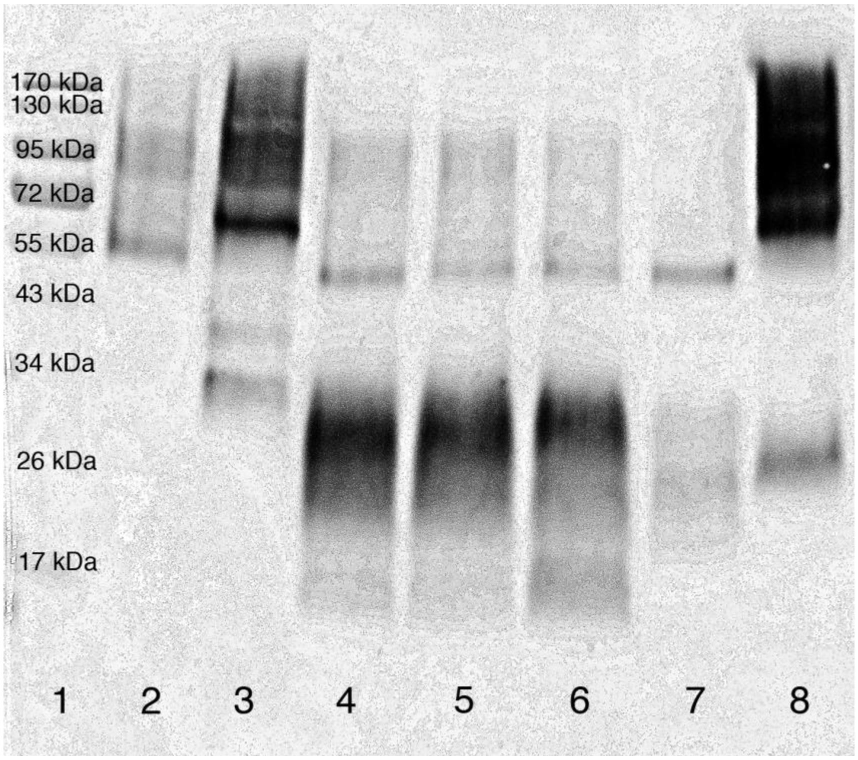

Supplementary Figure 14. Western blotting of enzymes secreted by *S. cerevisiae*. 20 µl of media supernatants of *S. cerevisiae* were analyzed by Western blotting of C-terminal Myc tag of lignin-degrading peroxidases. Lane 1, protein ladder (Fisher); lane 2, BA-vp; lane 3, CS-lip2; lane 4, PE-vpl1; lane 5, PE-vpl2; lane 6, PO-vp1; lane 7, PO-vp3; lane 8, TV-mrp. The expected molecular weight of the mature enzymes including affinity tags is between 37 and 38 kDa.

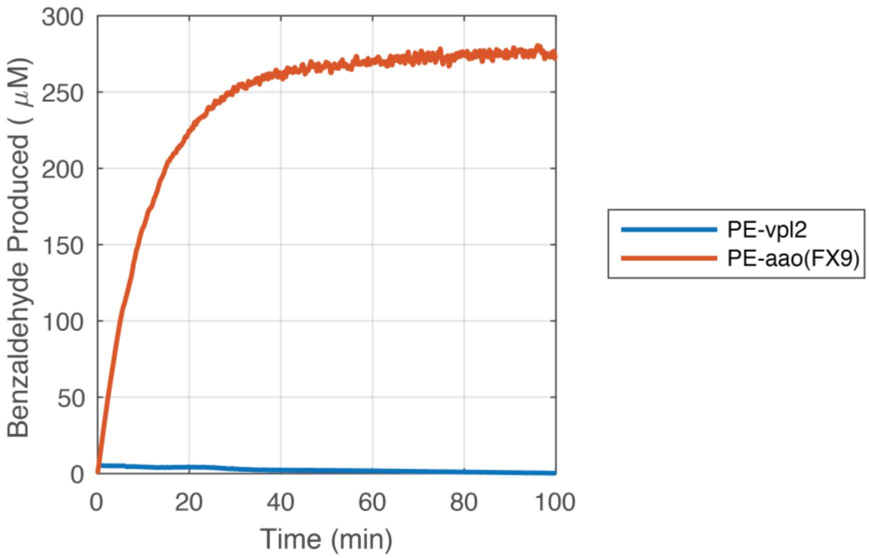

Supplementary Figure 15. PE-aao(FX9) and PE-vpl2 activities on benzyl alcohol as a substrate. Diafiltrated apoplast extracts of *N. benthamiana* expressing PE-aao(FX9)[10] or PE-vpl2 were incubated with 10 mM benzyl alcohol in 50 mM sodium tartrate, pH 3.5 and 100 μM H_2O_2 (only for PE-vpl2). Benzaldehyde production was measured spectroscopically at 250 nm (ε = 13800 1/M 1/cm)[11]. No benzyl alcohol activity was observed of PE-vpl2, whereas benzaldehyde was readily produced by PE-aao(FX9). PE-aao(FX9) activity decreases over time without full conversion of substrate presumably due to the enzyme's instability at the low pH of the assay.

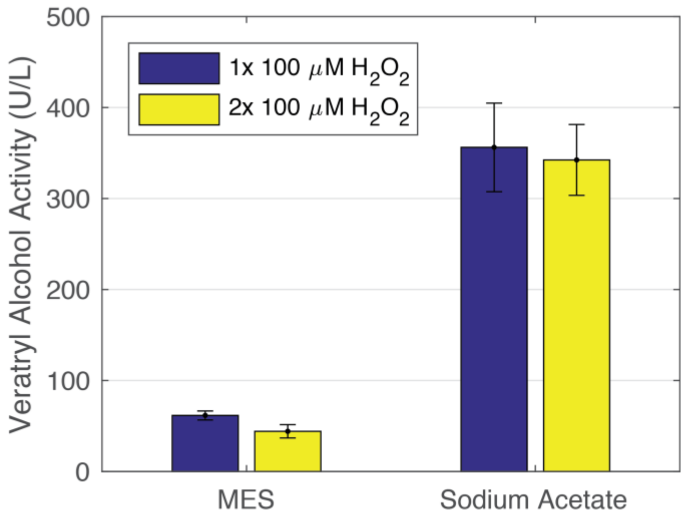

Supplementary Figure 16. Inhibitory effects of MES buffer on veratryl alcohol oxidation by commercial LiP. 0.16 mg/ml commercial lignin peroxidase (Sigma) was tested for activity against veratryl alcohol (2 mM) in the presence of peroxide (0.1 mM) and either 2-(N-morpholino)ethanesulfonic acid (MES) or sodium acetate buffer (13 mM, pH 6.0) using plate reader spectroscopy (see Methods). MES buffer inhibited LiP activity approximately six-fold compared to sodium acetate, and the latter was used for apoplast extraction of enzymes from *N. benthamiana*.

Supplementary Table 1. Heme concentration of diafiltrated extracts. Heme content was measured of diafiltrated apoplast extracts from *N. benthamiana* by the pyridine hemachromagen method[12] using absorbance at 557 nm and a molar extinction coefficient of 34700 1/M 1/cm.

**PC-*mnp1***	**PE-*vpl2***	**CS-*lip1***	**GFP**
5.00 µM	3.93 µM	4.23 µM	1.97 µM

Supplementary Table 2. List of strains used.

**Strain**	**Species**	**Genotype**	**Reference**
BJ5465	*S. cerevisiae*	MATa ura3-52 trp1 leu2-delta1 his3-delta200 pep4::HIS3 prb1-delta1.6R can1 GAL	Ref. 7
JHY693	*S. cerevisiae*	MATa his3Δ1 leu2Δ0 ura3Δ0 met15Δ0 SAL1+ HAP1+ CAT5(91M) MIP1(661T) MKT1(30G) RME1(INS-308A) TAO3(1493Q) prb1Δ pep4Δ	Ref. 1
GV3103	*A. tumefaciens*		

Supplementary Table 3. List of vectors used.

**Plasmid**	**Description**	**Reference**
pRS415-ADH2	Leu2-P_{ADH2}-MCS-T_{TEF1}-$ORI_{CEN/ARS}$-AmpR	Dr. Colin Harvey, SGTC
pCHINT2AL	Leu2-P_{ADH2}-flag-MCS-T_{TEF1}-$ORI_{2\mu}$-AmpR	[2]
pL131	Leu2-P_{ADH2}-αMF*appS4*-HA-MCS-Myc-T_{TEF1}-$ORI_{CEN/ARS}$-AmpR	This study
pL231	Leu2-P_{ADH2}-αMF*appS4*-HA-MCS-Myc-T_{TEF1}-$ORI_{2\mu}$-AmpR	This study
pEAQ	P_{35S}-5'UTR_{CPMV}-PhDIRSP-MCS-Myc-His6-3'UTR_{CPMV}-T_{NOS}-KanR	[13]

Supplementary Table 4. List of ER signal peptides used.

ER Signal Peptide	Protein Sequence	DNA Sequence
PhDIR	MGGEKAFSFIFLLFVCFFLANLSGSSA	atgggaggagaaaaagctttcagtttcattttcctcctcttcgtgtgcttcttcctagccaacctctctgggtcttcagct
NbDIR	MEKLNLILLLSSIAITISSIPFAHA	atggaaaagctaaacctaattctattgctttcctccattgctattaccatatcatcaattccgtttgctcatgcc
NbSEN4	MSCKLVLALMVSAFAIATA	atgtcttgtaaattagtactagctcttatggttagtgcttttgctattgcaactgcg
αMFappS4	MRFPSIFTAVVFAASSALAAPANTTAEDETAQIPAEAVIGYLGLEGDSDveratryl alcoholALPLSDSTNNGSLSTNTTIASIAAKEEGVSLDKREAEA	atgagatttccttcaatttttactgcagttgtattcgcagcatcctccgcattagctgctccagccaacactacagcagaagatgaaacggcacaaattccggctgaagctgtcatcggttacttaggtttagaaggggattccgatgttgctgctttgccattgtccgacagcacaaataacgggtcattgtctacaaatactactattgccagcattgctgctaaagaagaaggggtatctttggataaaagagaggctgaagct
SUC2	MLLQAFLFLLAGFAAKISA	atgcttttgcaagctttccttttccttttggctggttttgcagccaaaatatctgca
INU1	MKLAYSLLLPLAGVSASVINYKR	atgaagttagcatactccctcttgcttccattggcaggagtcagtgcttcagttatcaattacaagaga

pre-Ost1-pro-αMF	MRQVWFSWIVGLFLCFFNVSSAAPVNTTTEDETAQIPAEAVIGYLDLEGDFDveratryl alcoholVLPFSNSTNNGLLFINTTIASIAAKEEGVSLDKREAEA	atgaggcaggtttggttctcttggattgtgggattgttcctatgtttttcaacgtgtcttctgctgctccagtcaacactacaacagaagatgaaacggcacaaattccggctgaagctgtcatcggttacttagatttagaaggggatttcgatgttgctgttttgccattttccaacagcacaaataacgggttattgtttataaatactactattgccagcattgctgctaaagaagaaggggtatctttggataaaagagaggctgaagct
K28	MESVSSLFNIFSTIMVNYKSLVLALLSVSNLKYARG	atggaatccgtcagttccttgttcaacattttctccaccatcatggtcaactacaagtctttggttttggccttgttgtccgtttctaatttgaaatacgctagaggt
Kα	MNIFYIFLFLLSFVQGLEHTHRRGSLVKR	atgaatatattttacatatttttgtttttgctgtcattcgttcaaggtttggagcatactcatcgaagaggctccttagtcaaaagg
SCSP	MKVLIVLLAIFAALPLALAQPVISTTVGSAAEGSLDKREA	atgaaggttttgattgtcttgttggctatcttcgctgctttgccattggccttagctcaaccggttatttctactaccgtcggttccgctgcagaaggctctttggacaagagagaagct

Supplementary Table 5. List of antibody epitope tags used.

Tag	Protein Sequence	DNA Sequence
Human influenza hemagglutinin (HA)	YPYDVPDYA	tacccatacgacgttccagactacgct
c-Myc (Myc)	EQKLISEEDL	gaacaaaagcttatttctgaagaggacttg
StrepTag	WSHPQFEK	tggtctcatccacaatttgaaaaa
Myc-His6	ASEQKLISEEDLNSAVDHHHHHH	gctagcgaacaaaaactcatctcagaagaggatctgaatagcgccgtcgaccatcatcatcatcatcat

SI References:

1. Jones, E. W. Tackling the Protease Problem in Saccharomyces cerevisiae. *Methods Enzymol.* (1991). doi:10.1016/0076-6879(91)94034-A
2. Harvey, C. J. B. *et al.* HEx: A heterologous expression platform for the discovery of fungal natural products. *Sci. Adv.* (2018). doi:10.1126/sciadv.aar5459
3. Sikorski, R. S. & Hieter, P. A system of shuttle vectors and yeast host strains designed for efficient manipulation of DNA in Saccharomyces cerevisiae. *Genetics* **122**, 19–27 (1989).
4. Rakestraw, J. A., Sazinsky, S. L., Piatesi, A., Antipov, E. & Wittrup, K. D. Directed evolution of a secretory leader for the improved expression of heterologous proteins and full-length antibodies in Saccharomyces cerevisiae. *Biotechnol. Bioeng.* (2009). doi:10.1002/bit.22338
5. Fitzgerald, I. & Glick, B. S. Secretion of a foreign protein from budding yeasts is enhanced by cotranslational translocation and by suppression of vacuolar targeting. *Microb. Cell Fact.* (2014). doi:10.1186/s12934-014-0125-0
6. Huang, D. & Shusta, E. V. Secretion and surface display of green fluorescent protein using the yeast Saccharomyces cerevisiae. *Biotechnol. Prog.* (2005). doi:10.1021/bp0497482
7. Tien, M. & Kirk, T. K. Lignin peroxidase of Phanerochaete chrysosporium. *Methods Enzymol.* (1988). doi:10.1016/0076-6879(88)61025-1
8. O'Leary, B. M., Rico, A., McCraw, S., Fones, H. N. & Preston, G. M. The infiltration-centrifugation technique for extraction of apoplastic fluid from plant leaves using Phaseolus vulgaris as an example. *J. Vis. Exp.* (2014). doi:10.3791/52113
9. Bradford, M. M. A rapid and sensitive method for the quantitation of microgram quantities of protein utilizing the principle of protein-dye binding. *Anal. Biochem.* **72**, 248–54 (1976).
10. Viña-Gonzalez, J., Elbl, K., Ponte, X., Valero, F. & Alcalde, M. Functional expression of aryl-alcohol oxidase in Saccharomyces cerevisiae and Pichia pastoris by directed evolution. *Biotechnol. Bioeng.* (2018). doi:10.1002/bit.26585
11. GUILLÉN, F., MARTÍNEZ, A. T. & MARTÍNEZ, M. J. Substrate specificity and properties of the aryl-alcohol oxidase from the ligninolytic fungus Pleurotus eryngii. *Eur. J. Biochem.* (1992). doi:10.1111/j.1432-1033.1992.tb17326.x
12. Barr, I. & Guo, F. Pyridine Hemochromagen Assay for Determining the Concentration of Heme in Purified Protein Solutions. *BIO-PROTOCOL* (2015). doi:10.21769/bioprotoc.1594
13. Sainsbury, F., Thuenemann, E. C. & Lomonossoff, G. P. PEAQ: Versatile expression vectors for easy and quick transient expression of heterologous proteins in plants. *Plant Biotechnol. J.* (2009). doi:10.1111/j.1467-7652.2009.00434.x
