# Supplementary material for: A plant host, Nicotiana benthamiana, enables the production and study of fungal lignin-degrading enzymes
Source: Commun Biol. 2021 Sep 1;4:1027. doi: 10.1038/s42003-021-02464-9 (PMC8410833; doi:10.1038/s42003-021-02464-9)
Supplement: Supplementary file 2 — Description of Supplementary Files [file 42003_2021_2464_MOESM2_ESM.pdf]

Description of Additional Supplementary Files

File name: Supplementary Data 1

Description: List of genes and corresponding codon-optimized DNA sequences used in this study and source data for graphs and charts used in manuscript figures.
